# Common Environmental Responses of Bird Functional and Visual-Attractiveness Diversity in Urban Residential Areas

**DOI:** 10.3390/biology15211972

**Published:** 2026-11-02

**Authors:** Deng Pan, Yaqin Ding, Yujun Chen, Siteng Wu, Ziang Su, Juan Wang, Zhipeng Zhu, Yuxiang Liu, Xiaoling Xu, Weicong Fu

**Affiliations:** 1 College of Landscape Architecture and Art, Fujian Agriculture and Forestry University, Fuzhou 350002, China; 2 College of Architecture and Urban Planning, Fujian University of Technology, Fuzhou 350118, China; 3 Faculty of Forestry & Environmental Stewardship, University of British Columbia, Vancouver, BC V6T 1Z4, Canada

**Keywords:** urban residential areas, bird functional diversity, visual aesthetic attractiveness, common environmental responses, vegetation management

## Abstract

Urban residential areas are important habitats for birds and settings for residents’ daily contact with nature. Whether morphological functional-trait diversity and variation in species-level visual-attractiveness scores share environmental responses remains unclear. We surveyed 78 transects within 33 residential areas along the Min River in Fuzhou, China, over 12 months and integrated local vegetation, built-environment, and multi-scale landscape predictors. Grouped nested cross-validation, extreme gradient boosting (XGBoost), and out-of-fold (OOF) SHapley Additive exPlanations (SHAP) were used to compare predicted responses. Model performance was moderate for five metrics (OOF R^2^ = 0.250–0.404). (1) Total tree cover, mean tree height, and tree richness were the most consistent predictors of functional-trait and visual-attractiveness dispersion, while other effects were metric-specific. (2) Matched functional dispersion (FDis) and Rao’s quadratic entropy (Rao’s Q) metrics showed predicted responses that were predominantly concordant with those for tree-related variables, whereas local response divergence was concentrated in low-contribution understory variables. Most functional–visual associations and response-curve similarities did not consistently exceed null expectations from shared community composition. Model performance also declined under square-root and presence/absence weighting. (3) These findings support consideration of mature-tree retention, context-appropriate canopy structure, tree richness, and differentiated understory management, without treating higher-response intervals as causal targets. Concordance does not establish independent ecological synergy or measured aesthetic benefits.

## Simple Summary

Urban residential areas provide important habitats for birds and are also places where people frequently encounter wildlife in daily life. We examined whether bird communities with diverse morphological traits and varied species-level visual-attractiveness scores respond similarly to environmental conditions. Birds were surveyed monthly along 78 transects in 33 residential areas in Fuzhou, China, while local vegetation structure was measured in the field. Tree cover, tree height, and the number of tree species were the most consistent predictors of functional-trait diversity and variation in visual-attractiveness scores. These two dimensions often showed similar predicted responses to major tree-related conditions, while localized differences occurred mainly for less influential shrub and herbaceous variables. However, most associations and response-curve similarities did not consistently exceed expectations arising from the shared bird communities. Results also varied when bird observations were weighted differently. The findings suggest that parties responsible for residential vegetation management should consider retaining mature trees, maintaining tree-species diversity, and adapting understory vegetation to local conditions. They do not establish independent ecological synergy or measured improvements in residents’ visual experiences.

## 1. Introduction

Urban birds are a prominent component of people’s everyday contact with nature [1]. They contribute to urban ecosystem functioning through pest control, seed dispersal, and other ecological functions [2], while plumage coloration, ornamentation, and other visual traits shape people’s aesthetic appreciation of birds [3,4]. Together, these ecological and aesthetic dimensions represent complementary aspects of the values that urban bird communities provide to people. Residential areas and their surrounding neighborhoods are particularly important settings for such everyday encounters, where birds are among the most readily observed wildlife [5,6]. Focusing on a single dimension may therefore overlook part of the broader value of bird communities [7]. Recent trait-based research has also examined the potential contributions of urban bird communities to both regulating and cultural ecosystem services [8]. Morphological functional-trait diversity describes variation in selected bird traits, not directly measured ecosystem functioning. Likewise, variation in species-level visual-attractiveness scores should not be equated with residents’ aesthetic experiences.

Research on urban birds has increasingly extended beyond citywide urbanization gradients and large green spaces, such as parks, to residential environments where people frequently encounter birds in daily life. Research has also broadened from describing species composition to examining how environmental conditions shape multiple attributes of bird communities [9,10]. Vegetation composition, blue–green spaces, the built environment, and surrounding landscapes can all influence bird communities in residential areas [11,12]. Within this broader framework, functional-trait diversity characterizes variation in morphological attributes such as body size, bill morphology, and flight-related morphology, which may reflect environmental filtering of community composition. Intense urbanization can reorganize bird functional composition and reduce functional diversity [13]. Bird responses to environmental gradients may be nonlinear, and interpretable machine-learning approaches can help characterize patterns that are difficult to represent using conventional linear models [14].

In parallel with this ecological perspective, increasing attention has been given to how people perceive the appearance of birds. Plumage coloration, ornamentation, and other visual traits can influence aesthetic evaluations of bird species [3,4]. Earlier studies often relied on questionnaires administered to residents or birdwatchers, whereas standardized datasets such as iRateBirds now enable visual aesthetic attractiveness to be quantified consistently across species [15], providing a basis for community-level assessment. At the community level, mean and community-weighted mean scores summarize attractiveness levels, whereas visual functional dispersion (visual FDis), visual Rao’s quadratic entropy (visual Rao’s Q), and score range describe variation among co-occurring species. Greater dispersion does not necessarily indicate higher overall attractiveness or greater cultural benefits experienced by residents. Research on landscape preference and the visual quality of residential green spaces has also emphasized the importance of human perception and landscape context in evaluating aesthetic experiences [16,17]. Visual aesthetic appreciation represents one component of the broader cultural values associated with birds, and previous studies have reported relationships between the ecological and cultural dimensions of bird communities [18,19,20]. Environmental conditions can also simultaneously influence bird attributes associated with ecological functions and cultural services, including visual aesthetic appeal [19]. Because functional and visual indices are calculated from the same bird assemblages, apparent concordance may partly reflect shared community composition and metric construction. However, whether bird communities exhibit concordant responses in functional diversity and visual-attractiveness diversity along the same environmental gradients remains unclear, particularly in urban residential areas.

Building on these insights, we examined 33 urban residential areas in Fuzhou, China, using variables describing internal vegetation structure and the built environment, together with surrounding-landscape variables measured at multiple spatial scales. We considered functional-trait and visual-attractiveness diversity as distinct community attributes, rather than as direct measures of ecosystem functioning or aesthetic benefits experienced by residents. We defined similar model-predicted changes in matched functional and visual metrics along the same environmental gradient as common environmental responses and localized changes in opposite directions as local response divergence, rather than as demonstrated ecological trade-offs. Based on previous evidence showing that vegetation structure shapes urban bird community composition, we expected local vegetation structure, particularly tree-related variables, to contribute strongly to both dimensions, while the degree of concordance between functional and visual responses remained an empirical question. Our objectives were to (1) identify environmental factors associated with bird functional diversity and visual-attractiveness diversity and characterize their response patterns; (2) determine whether matched functional and visual metrics show concordant or divergent responses along major environmental gradients and assess whether observed concordance exceeds expectations arising from shared community composition; and (3) discuss potential vegetation-management implications based on these response patterns. By integrating functional and visual aesthetic metrics within a common predictive framework, this study evaluates whether the same environmental conditions are associated with concordant responses across both dimensions and offers context-dependent implications for vegetation management in urban residential areas.

## 2. Materials and Methods

### 2.1. Study Area

**Figure 1:**
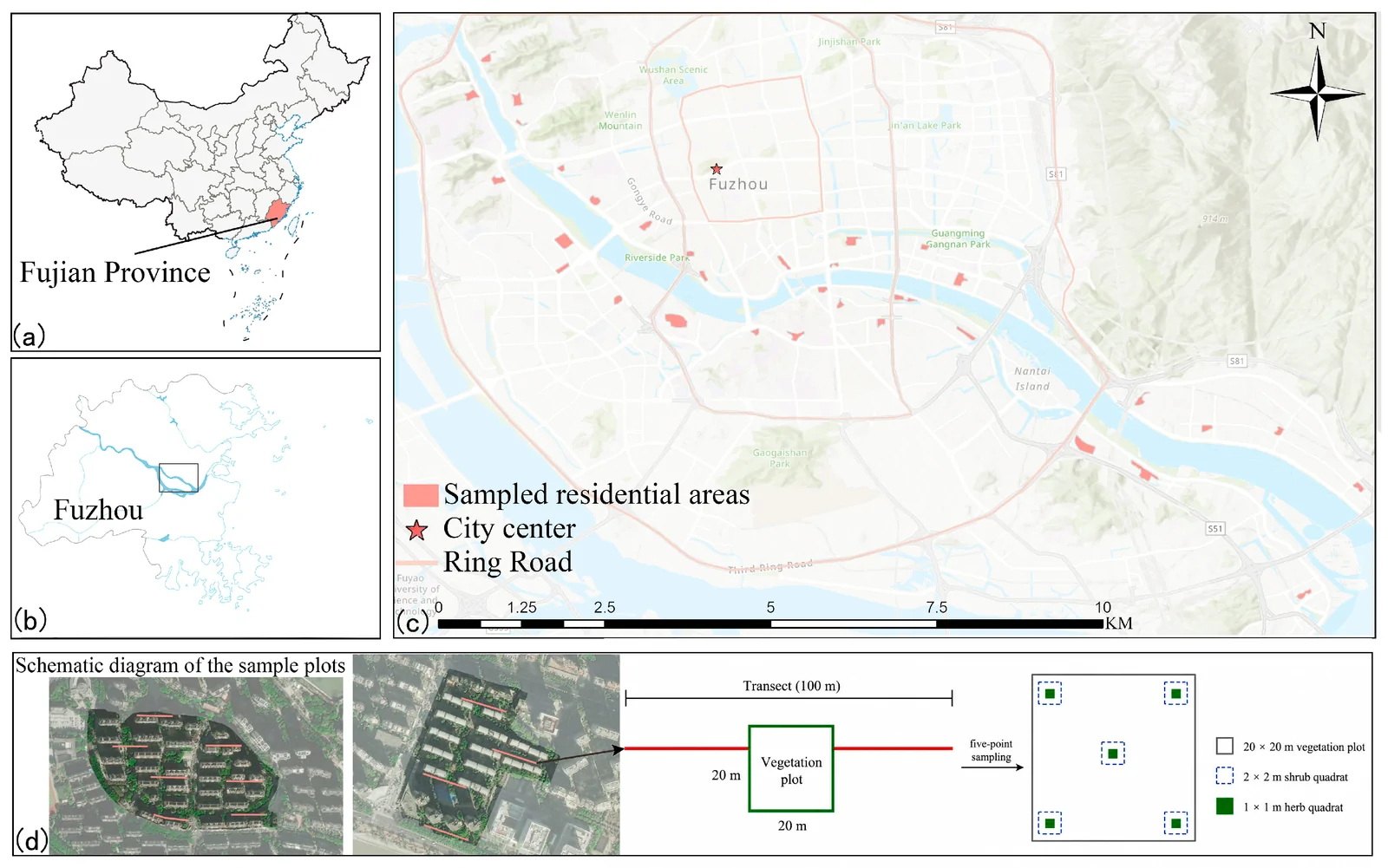
Study area and sampling design in Fuzhou, China. (**a**–**c**) Regional location and distribution of the 33 sampled residential areas; (**d**) examples of sampled sites and the 100-m transect with nested vegetation plots.

The study area is located along the lower reaches of the Min River in central Fuzhou, Southeastern China (26°00′–26°09′ N, 119°24′–119°42′ E). The Min River forms an important axis of urban development in Fuzhou, and the Fuzhou Territorial Spatial Master Plan (2021–2035) identifies an along-river development axis as a major component of the city’s spatial structure [21]. We selected residential areas along the Min River corridor to capture variation in construction period, building form, vegetation structure, and surrounding landscape while limiting broad-scale climatic and river-valley variation among sites. Within a 1-km buffer around the Min River centerline, we selected 33 distinct residential-area polygons delineated using boundary data from the Baidu Maps Open Platform, each covering at least 2 ha. The 2-ha threshold was adopted as an operational site-selection criterion informed by [22], rather than as an empirically established minimum habitat area for urban birds. Figure 1 shows the study area and sampling design, including the distribution of sampled residential areas and the transect and nested vegetation plots.

### 2.2. Data Collection

#### 2.2.1. Transect Setup and Bird Surveys

We established approximately one 100-m transect per 2 ha of residential area, with at least 100 m separating adjacent transects. The final design comprised 78 transects across 33 residential areas (Figure 1d). Each transect was surveyed once per month from October 2023 through September 2024, yielding 12 survey rounds. Surveys were conducted within 4 h of sunrise under clear weather conditions and in the absence of strong winds. Visit times were varied among seasonal rounds where feasible. Two researchers surveyed each transect together, one primarily identifying birds and the other recording species and counts. Birds seen or heard within 25 m on either side of each transect were recorded, with binoculars used when needed. Observers walked each transect at approximately 3 km/h between bird observations, pausing as needed for identification and counting. Each visit generally lasted 5–20 min. Records that could not be confidently identified in the field were subsequently verified using photographs, audio recordings, and expert consultation. Bird nomenclature and taxonomy followed A Checklist on the Classification and Distribution of the Birds of China (4th ed.) [23].

#### 2.2.2. Plant Community Survey

Plant communities were surveyed once in spring 2024. At the midpoint of each bird transect, we established one 20 m × 20 m tree plot, five 2 m × 2 m shrub subplots, and five 1 m × 1 m herbaceous subplots arranged in a five-point sampling design. Where site constraints prevented the standard layout, the plot configuration was adjusted while maintaining the specified sampling areas. We recorded species composition and vegetation structure for each vegetation layer. Tree height and first-branch height were visually estimated, whereas diameter at breast height and east–west and north–south crown widths were measured using measuring tape. Total tree cover was calculated from the vertical projections of tree crowns within the plot, with overlapping areas counted only once. Shrub and herbaceous cover were determined from tape-measured vegetation coverage within their respective subplots. From these data, we calculated mean tree height, total tree cover, tree richness, total shrub cover, shrub richness, total herbaceous cover, and herbaceous richness.

#### 2.2.3. Built Environment and Surrounding Landscape Data

Building footprint and floor-count data were obtained from the Urban Data Analyst Platform [24], and construction year data were obtained from 58 Anjuke [25]. We calculated residential area size, building density, floor area ratio, and mean building height using ArcGIS Pro 3.0.1 (Esri, Redlands, CA, USA). We then created 300-, 500-, 1000-, and 1500-m buffers based on the boundaries of the 33 residential areas. Within the surrounding buffer areas, excluding the corresponding residential-area polygons, we extracted land-cover variables [26], vegetation status [27], and roads [28]. Detailed predictors and data sources are provided in Table S1 and Supplementary Methods SM5.

### 2.3. Data Processing and Research Methods

#### 2.3.1. Bird Community Matrix

The 12 rounds of bird surveys were aggregated by transect and species to construct an annual transect × species community matrix. For each transect, the cumulative annual number of observations of each species was used to represent its relative observational weight under standardized survey effort. We divided the cumulative count of each species by the total number of bird observations within the same transect to obtain species-level proportions. These proportions were subsequently used as weights in the calculation of observation-weighted functional and visual metrics. These proportions represent relative observational frequencies and do not account for species-specific differences in detection probability. Alternative weighting and seasonal sensitivity analyses are described in Supplementary Methods SM2, with results summarized in Table S5.

#### 2.3.2. Bird Functional Traits and Functional Diversity

Functional trait data were obtained from AVONET [29], with trait data available for all 56 bird species recorded in this study. Seven continuous morphological traits were selected: bill length, bill width, bill depth, tarsus length, tail length, body mass, and hand-wing index (HWI). The six positive morphological measurements were log10-transformed, while HWI was retained on its original scale; all seven traits were then z-score standardized before principal component analysis (PCA). The first two principal components explained 82.56% (PC1: 67.48%; PC2: 15.08%) of the total trait variance and were used to define a two-dimensional functional trait space (Table S3). PC1 mainly represented overall morphological size, whereas PC2 was primarily associated with HWI. The resulting PC scores provided species coordinates for calculating functional richness (FRic) and the Euclidean distances underlying functional dispersion (FDis) and Rao’s quadratic entropy (Rao’s Q). Based on this trait space, we calculated FRic, FDis, and Rao’s Q. FRic represents the extent of functional trait space occupied by the community [30]. FDis represents the observation-weighted dispersion of species around the community centroid in functional trait space [31], whereas Rao’s Q integrates pairwise functional distances among species weighted by their relative observational proportions [32]. The sensitivity of FDis and Rao’s Q to the functional trait-space representation was further evaluated using all seven standardized morphological traits (Supplementary Methods SM2; Table S6).

#### 2.3.3. Visual-Attractiveness Metrics of Bird Communities

Species-level visual aesthetic attractiveness scores were obtained from the iRateBirds citizen-science dataset [15]. The dataset contains 408,207 valid ratings from 6212 participants, who evaluated bird photographs from the Macaulay Library on a scale from 1 to 10. The iRateBirds dataset provides three visual-attractiveness measures: raw mean ratings, full-model predictions adjusted for photograph quality and user language, and subset-model predictions that additionally account for respondent-specific characteristics. We used the species-level full-model predictions (predicted_attractiveness_full_model), which also account for variation among photographs, bird species, and bird families. The dataset provides visual aesthetic attractiveness scores for 11,319 taxonomic units, and all 56 bird species recorded in this study were successfully matched.

Let A_i_ denote the full-model visual aesthetic attractiveness score of species i, S_j_ the number of bird species recorded in transect j, and p_ij_ the relative observational proportion of species i in transect j. The mean visual aesthetic attractiveness of the bird community was calculated as follows:

$${\bar{A}}_{j}=\frac{1}{S_{j}}\sum_{i=1}^{S_{j}}A_{i}$$

The community-weighted mean (CWM) visual aesthetic attractiveness was calculated as follows:

$${\mathrm{CWM}}_{j}=\sum_{i=1}^{S_{j}}p_{\mathrm{ij}}A_{i}$$

Visual FDis was calculated in a one-dimensional visual aesthetic attractiveness space following the definition of functional dispersion [31]:

$${\mathrm{VFDis}}_{j}=\sum_{i=1}^{S_{j}}p_{\mathrm{ij}}\left|A_{i}-{\mathrm{CWM}}_{j}\right|$$

Visual Rao’s quadratic entropy (visual Rao’s Q) was calculated using the absolute differences in visual aesthetic attractiveness scores among species [32]:

$${\mathrm{VQ}}_{j}=\sum_{i=1}^{S_{j}}\sum_{k=1}^{S_{j}}p_{\mathrm{ij}}p_{\mathrm{kj}}\left|A_{i}-A_{k}\right|$$

VFDis_j_ represents the observation-weighted dispersion of species’ visual aesthetic attractiveness scores around the community-weighted mean, whereas VQ_j_ represents the expected absolute difference in species-level visual-attractiveness scores between two independent draws based on the relative observational weights of the recorded species. Visual range was calculated as the difference between the highest and lowest species-level attractiveness scores recorded within each transect.

Mean visual attractiveness and visual CWM characterize the central tendency of species-level attractiveness scores, whereas visual FDis and visual Rao’s Q characterize within-community variation in these scores. These metrics characterize the distribution of standardized species-level visual-attractiveness scores rather than aesthetic experiences directly reported by local residents.

### 2.4. Statistical Analysis

**Figure 2:**
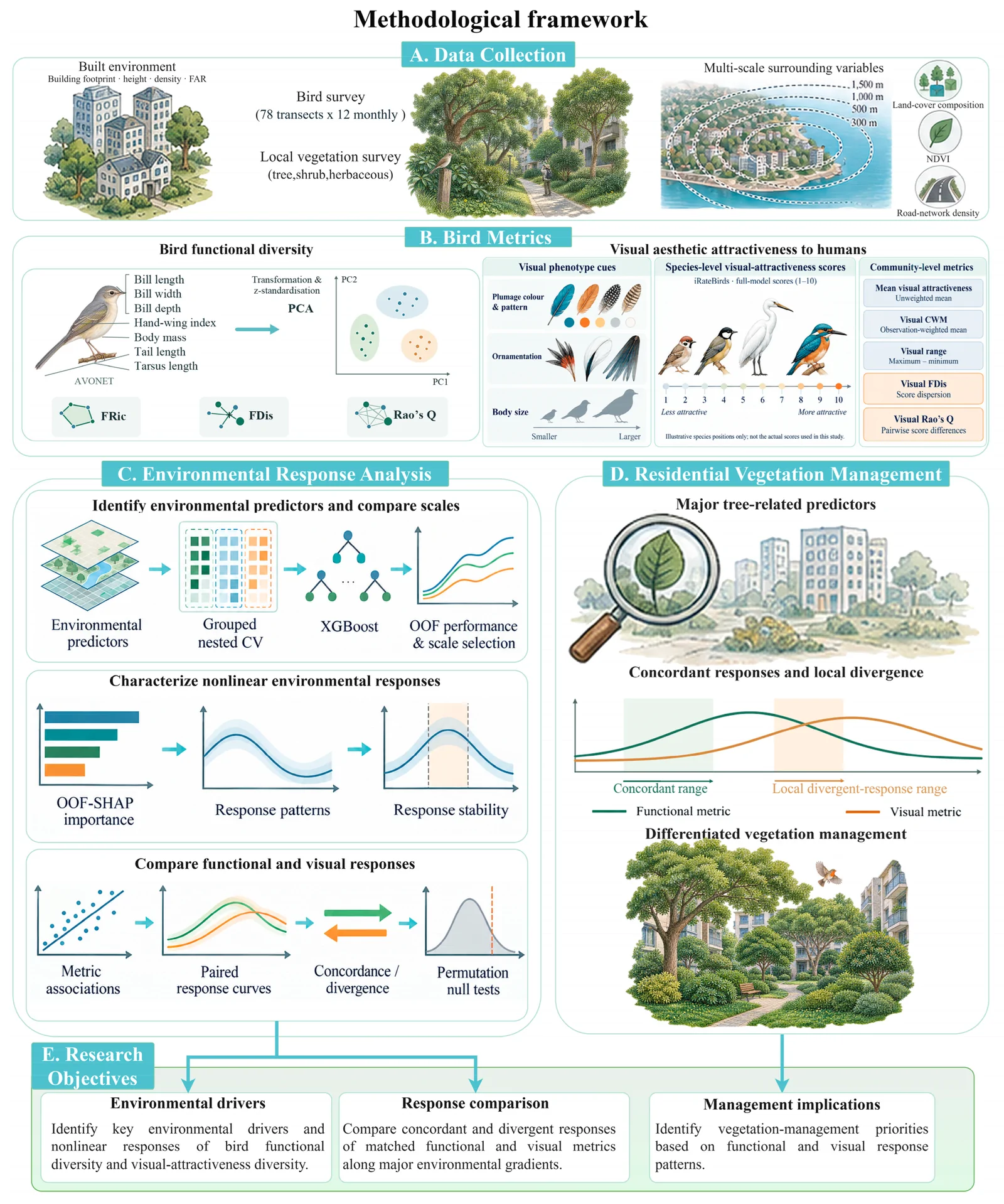
Methodological framework of the study. FAR, floor area ratio; NDVI, normalized difference vegetation index; PCA, principal component analysis; PC, principal component; FRic, functional richness; FDis, functional dispersion; Rao’s Q, Rao’s quadratic entropy; CWM, community-weighted mean; CV, cross-validation; XGBoost, extreme gradient boosting; OOF, out-of-fold; SHAP, SHapley Additive exPlanations.

Using the 78 transects as the units of analysis, we conducted three sets of analyses: model prediction and variable screening, response interpretation, and comparison of response metrics. The overall analytical framework is summarized in Figure 2. Transects were retained as the analytical units to characterize within-residential-area variation in vegetation conditions, while residential-area identifiers were used for grouped model validation to account for the nested sampling design.

(1) Model prediction and variable screening were used to identify key environmental predictors and compare out-of-sample predictive performance across spatial scales. We fitted extreme gradient boosting (XGBoost) models [33] within a repeated nested cross-validation framework grouped by residential area [34]. Before model fitting, we checked the training data for missing values and near-zero-variance predictors and screened correlated predictors using an absolute Spearman correlation threshold of |ρ| = 0.85. For each response metric, we compared an internal-environment model with candidate models that additionally included surrounding-landscape variables at 300, 500, 1000, or 1500 m. The outer loop comprised five folds repeated three times to generate out-of-fold (OOF) predictions, whereas the four-fold inner loop was used for spatial-scale and hyperparameter selection. Predictive performance was evaluated using OOF R^2^, root mean square error (RMSE), mean absolute error (MAE), and Spearman’s ρ. Response metrics with weak predictive performance were not carried forward to subsequent environmental-response analyses but were retained for descriptive and association analyses. The predictor-screening criteria, hyperparameter search strategy, and grouped nested cross-validation procedures are described in Supplementary Methods SM1, while complete candidate parameter settings and random-seed configurations are retained in the accompanying analysis scripts.
(2) SHapley Additive exPlanations (SHAP) were used to quantify predictor contributions and characterize response direction, nonlinear patterns, and turning-point intervals. For stable nonlinear responses, turning points were used to delineate higher-response ranges. SHAP values were calculated only for observations in the outer test folds, and variable importance was quantified as the mean absolute SHAP value [35,36]. The stability of response patterns was assessed using cluster bootstrap resampling at the residential-area level, and the main turning points of stable unimodal or U-shaped responses were identified from smoothed response curves. SHAP was used solely to interpret model predictions and was not treated as evidence of statistical significance or causality. Uncertainty in binned SHAP summaries, response-shape stability, and predictor rankings was assessed separately using residential-area cluster bootstrap (Supplementary Methods SM3; Table S7).
(3) Response comparisons were used to assess associations between functional and visual metrics and the concordance of their responses along the same environmental gradients. We calculated Spearman and partial Spearman correlations, with the latter controlling for bird species richness and cumulative bird observation counts. The robustness of these associations was assessed using cluster bootstrap resampling by residential area. We then compared stable SHAP responses of matched functional and visual metrics using curve correlations, concordant and opposing response intervals, and joint relative contributions to classify common environmental responses and local response divergence. Rule-sensitivity and leave-one-residential-area-out analyses were used to evaluate the stability of these classifications. Finally, two permutation-based null analyses were used to assess whether the observed functional–visual concordance could arise from shared community composition. Frequency-constrained permutations of species-level visual scores (4999 iterations) were used to generate null distributions for raw and partial functional–visual correlations, whereas a conditional XGBoost null model (199 permutations) was used to generate null intervals for matched response-curve correlations and joint relative importance. The conditional XGBoost null model retained the previously selected model configurations rather than repeating the full nested optimization procedure. Functional reference curves and the selected visual response-curve representations were held fixed. Observed and permuted visual responses were evaluated using the same linear or three-degree-of-freedom natural-spline representation for each target on a common 121-point predictor grid. Detailed permutation protocols and results are provided in Supplementary Methods SM4 and Tables S4 and S9. Residual spatial autocorrelation was assessed at the residential-area level using alternative nearest-neighbor definitions (Supplementary Methods SM5; Table S8).

All statistical analyses were conducted in R version 4.4.1. XGBoost models were fitted using the xgboost package (version 3.2.1.1), and SHAP results were summarized and visualized using the shapviz package (version 0.10.3). Analysis scripts, model inputs, and selected model outputs are provided in the accompanying Supplementary Data and Code archive, as described in the Data Availability Statement.

Selected illustrative elements in the methodological framework (Figure 2) were generated with assistance from OpenAI ChatGPT (GPT-5.6 Sol, 2026) and subsequently reviewed, edited, and verified by the authors. These elements are schematic only and do not represent research data or results.

## 3. Results

### 3.1. Bird Community and Vegetation Characteristics in Residential Areas

Across 78 transects in 33 residential areas, 12 monthly surveys recorded 56 bird species belonging to seven orders, 28 families, and 45 genera, yielding a cumulative total of 11,100 bird observations. The five most frequently recorded species were Eurasian Tree Sparrow, Swinhoe’s white-eye, the light-vented bulbul, the Chinese Blackbird, and the Spotted Dove. Forty species were residents, representing 71.43% of all recorded species. Vegetation surveys recorded 256 plant species belonging to 81 families and 200 genera, with woody species predominating overall. Common trees included camphor trees, Ficus species, and sweet osmanthus; the shrub layer was dominated by ivy trees, golden dewdrop, and the Chinese fringe flower; and creeping woodsorrel and Bermuda grass were common in the herbaceous layer.

Bird functional diversity and visual aesthetic attractiveness varied among transects (Table 1). Mean visual aesthetic attractiveness and the community-weighted mean (CWM) showed relatively narrow ranges, whereas the remaining functional and visual diversity metrics exhibited greater relative variation among transects.

### 3.2. Environmental Responses of Bird Functional Diversity and Visual-Attractiveness Metrics

#### 3.2.1. Environmental Responses of Bird Functional Diversity

**Figure 3:**
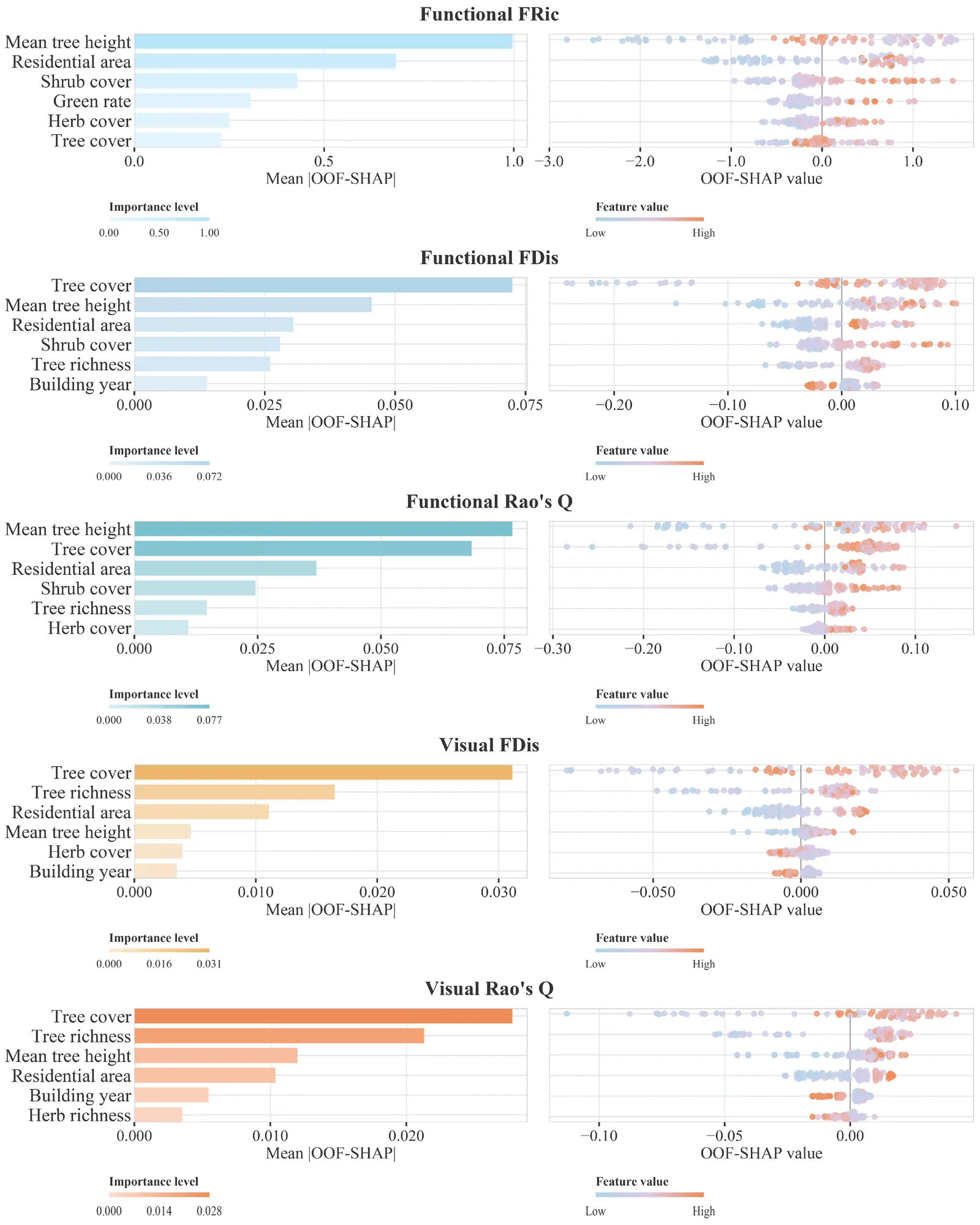
OOF-SHAP importance and contribution distributions for the five core response metrics. Bars show mean absolute OOF-SHAP values, and beeswarm points show transect-level OOF-SHAP contributions colored by predictor value.

All three functional diversity metrics showed positive out-of-sample predictive performance, with OOF R^2^ values of 0.250 for FRic, 0.364 for FDis, and 0.309 for Rao’s Q (Figure S1; Table S2). For each metric, models containing only internal-environment variables outperformed models incorporating surrounding-landscape variables, which provided no consistent improvement in predictive performance (Figure S2). Predictor importance was concentrated in tree structure, residential area size, and several shrub variables. Mean tree height and residential area size were the most important predictors of FRic, whereas total tree cover, mean tree height, and tree richness were the main predictors of FDis and Rao’s Q (Figure 3).

**Figure 4:**
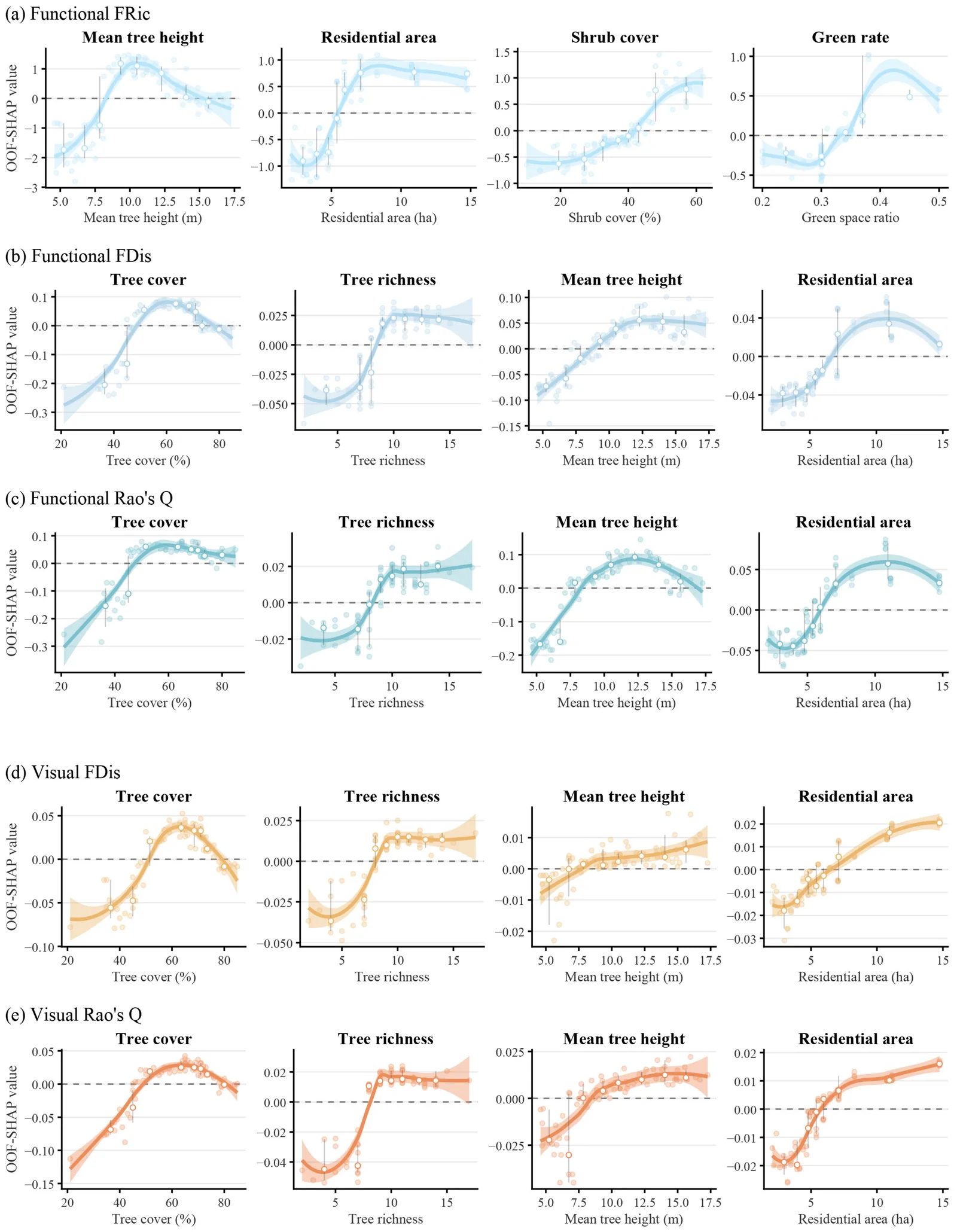
OOF-SHAP responses of the five core metrics to major environmental predictors. Points represent transect-level OOF-SHAP values, and solid curves show response patterns obtained using locally estimated scatterplot smoothing (LOESS). Shaded bands indicate approximate 95% pointwise intervals associated with the LOESS smoothers. Open circles represent binned median SHAP contributions, and error bars show their corresponding 95% percentile intervals from 400 residential-area cluster-bootstrap resamples. Neither interval accounts for uncertainty from refitting the XGBoost models.

Several major vegetation predictors showed pronounced nonlinear SHAP response patterns. Higher-response ranges for FRic occurred at mean tree heights of approximately 11–12 m and residential area sizes of approximately 10 ha. FDis and Rao’s Q responded similarly to total tree cover and mean tree height, with higher-response ranges at approximately 60–70% tree cover and 11–14 m mean tree height. SHAP values for FDis generally increased with tree richness and were relatively high at around 12 tree species (Figure 4). Total shrub cover showed positive SHAP responses for some functional metrics, whereas other understory variables, most other built-environment variables, and green rate showed relatively low contributions or limited response stability (Figures S3–S5). The stability of key response patterns, turning locations, and predictor rankings is summarized in Table S7.

#### 3.2.2. Environmental Responses of Visual-Attractiveness Metrics

Out-of-sample predictive performance varied markedly among the visual metrics. Visual FDis and visual Rao’s Q showed positive out-of-sample predictive performance, with OOF R^2^ values of 0.375 and 0.404, respectively. Mean visual aesthetic attractiveness, visual CWM, and visual range did not show consistently positive predictive performance. Subsequent environmental-response analyses therefore focused on visual FDis and visual Rao’s Q (Figure S1; Table S2). Predictor importance for both metrics was concentrated in tree-related vegetation variables. Total tree cover, tree richness, and mean tree height were among the most important predictors, while residential area size also contributed to model predictions (Figure 3).

Visual FDis and visual Rao’s Q showed similar nonlinear response patterns to total tree cover and tree richness. For both metrics, SHAP values initially increased and then leveled off or declined, with higher-response ranges at approximately 60–70% total tree cover and tree richness of 10–13 species. SHAP values also generally increased with mean tree height (Figure 4). Visual FDis additionally responded to total herbaceous cover and building year. SHAP values for total herbaceous cover increased to relatively high levels at approximately 30% cover and then declined, whereas building year showed an overall negative response. For visual Rao’s Q, herbaceous richness also showed an overall negative response. Other built-environment and understory variables showed relatively low importance or limited response stability (Figures S6 and S7). Additional weighting and community-representation analyses showed lower predictive performance under square-root and presence/absence weighting, whereas resident-only models performed similarly to the original annual models (Table S5; Figure S12).

### 3.3. Common Environmental Responses and Local Response Divergence Between Bird Functional Diversity and Visual-Attractiveness Diversity

#### 3.3.1. Associations Between Bird Functional and Visual Metrics

**Figure 5:**
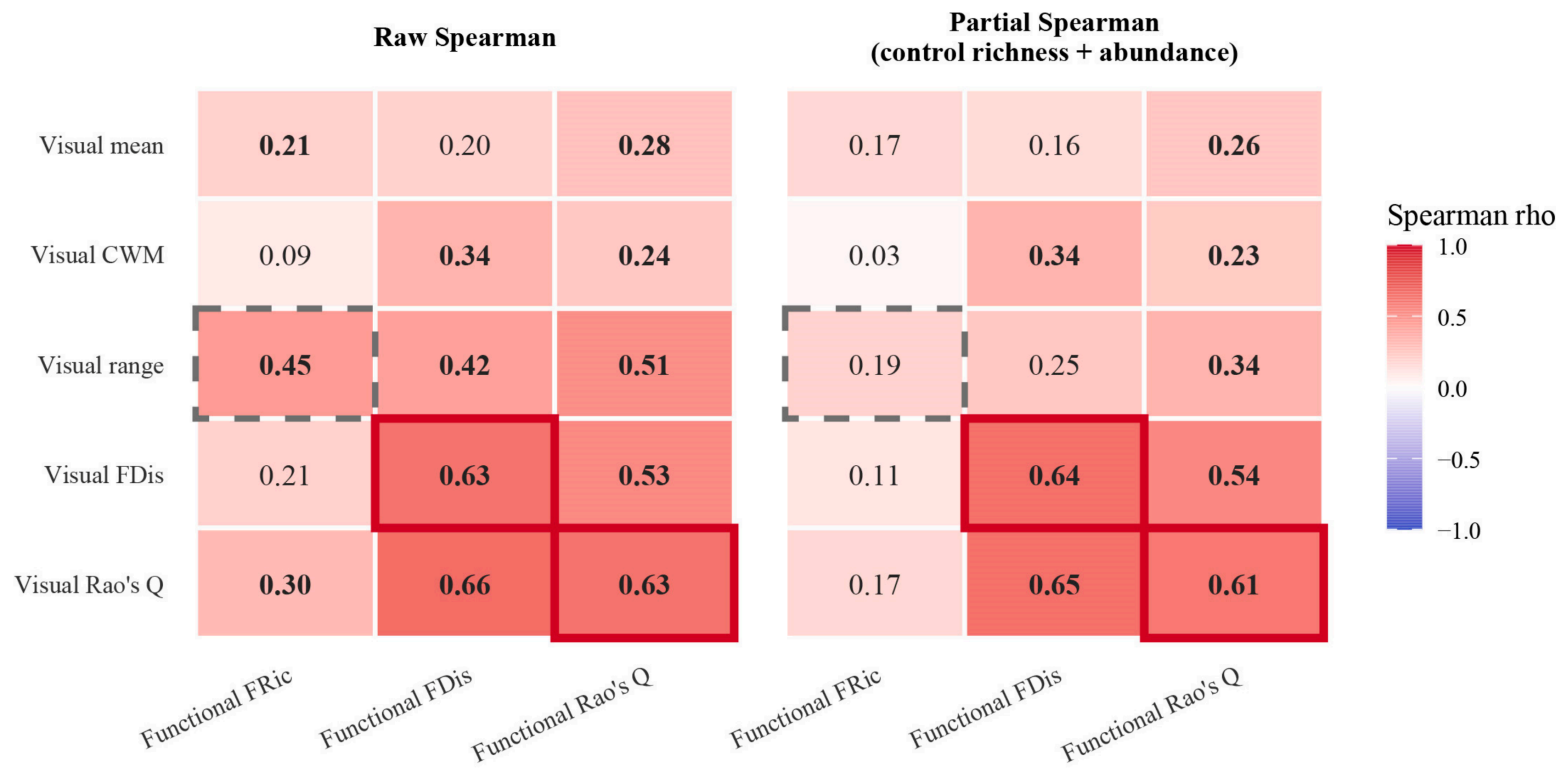
Associations between bird functional diversity and visual-attractiveness metrics. The left panel shows raw Spearman correlations, and the right panel shows partial Spearman correlations controlling for bird species richness and cumulative bird observation counts. Dashed outlines highlight the association between functional FRic and visual range, whereas red outlines indicate the matched functional–visual FDis and Rao’s Q comparisons.

Bird functional diversity and visual-attractiveness metrics were generally positively associated across transects (Figure 5). The raw and partial Spearman correlations between functional FDis and visual FDis were 0.627 and 0.643, respectively, while the corresponding correlations between functional and visual Rao’s Q were 0.632 and 0.612. By contrast, the partial correlation between functional FRic and visual range was substantially lower, at 0.194 (Figure S8). These associations generally weakened under square-root and presence/absence weighting, while resident-only and seasonal representations retained positive associations (Table S5).

#### 3.3.2. Concordant Environmental Responses and Local Response Divergence Between Matched Functional and Visual Metrics

**Figure 6:**
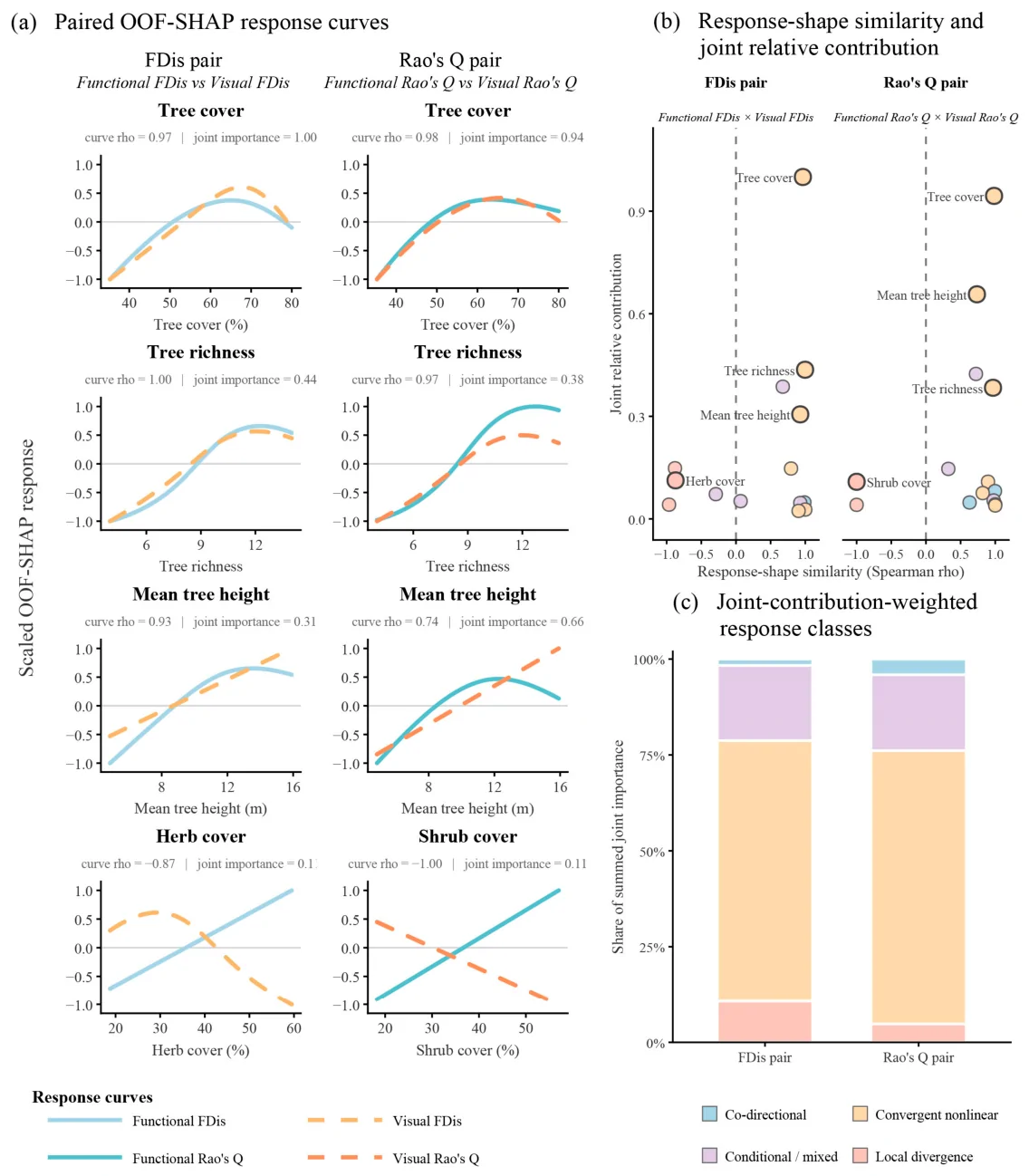
Common environmental responses and local divergences between matched functional and visual metrics. (**a**) Paired OOF-SHAP response curves; (**b**) response-curve similarity and joint relative contribution; (**c**) joint-contribution-weighted proportions of response classes.

Matched functional and visual metrics showed consistent responses to major tree-related gradients (Figure 6a,b). Functional FDis and visual FDis were highly correlated in their response curves for total tree cover, tree richness, and mean tree height (ρ = 0.928–0.997), with total tree cover showing the highest joint relative contribution. Both FDis metrics increased concordantly at approximately 35–64% tree cover and declined above approximately 68%. Concordant positive responses also occurred as tree richness increased from approximately 4 to 12 species and across mean tree heights of approximately 5–13 m. The paired Rao’s Q metrics showed similar patterns, particularly for total tree cover (ρ = 0.983; joint relative contribution = 0.945).

A small number of vegetation variables showed opposing responses. Functional and visual FDis responded oppositely to total herbaceous cover (ρ = −0.872), while the paired Rao’s Q metrics diverged in response to total shrub cover. However, the joint contribution of shrub cover was low (0.109) relative to total tree cover (0.945). Other differences, including those for greening rate, also had low joint contributions. Overall, concordant nonlinear responses remained dominant for both matched metric pairs (Figure 6c and Figure S9). The permutation-based null analyses indicated that most observed functional–visual associations and response-curve similarities did not consistently exceed expectations generated by shared community composition (Figure S10; Table S4). For four linearly represented response-curve comparisons, all 199 randomized absolute correlations equalled the observed magnitude (*p* = 1.000), indicating limited discriminatory ability of the absolute-tail tests (Table S9b). Nevertheless, responses of both matched pairs to total tree cover remained stable across alternative classification rules and leave-one-residential-area-out analyses, whereas some low-contribution variables were more sensitive to classification criteria (Figure S11).

## 4. Discussion

### 4.1. Environmental Responses of Bird Functional Diversity and Visual-Attractiveness Metrics in Residential Areas

This study showed that internal vegetation structure provided the most consistent predictive information for both bird functional diversity and visual-attractiveness metrics within residential areas. Although residential area size was important for FRic, most built-environment variables contributed less, and adding surrounding-landscape variables did not consistently improve out-of-sample predictive performance. Total tree cover, mean tree height, and tree richness ranked among the most important predictors across several functional and visual metrics. This pattern is consistent with previous studies showing that local vegetation structure can influence urban bird functional traits and functional diversity [37,38]. At broader spatial scales, however, neighborhood tree canopy, impervious surface, and landscape structure can shape bird communities in residential areas and the distribution of traits associated with bird attractiveness [19,39,40]. Thus, our results do not indicate that surrounding landscapes are ecologically unimportant; rather, within the environmental gradients and spatial scales represented in this study, internal vegetation structure provided more stable predictive information. The restricted Min River corridor sampling frame may also have limited variation in surrounding-landscape conditions.

Within this overall pattern, individual metrics differed in their responses to vegetation structure. FRic reflects the extent of functional trait space occupied by a community and responded most strongly to mean tree height and residential area size. In contrast, FDis and Rao’s Q, which characterize the dispersion and pairwise differences of functional traits, respectively, were more strongly associated with total tree cover, mean tree height, and tree richness. Greater vertical and compositional complexity can increase the range of foraging, perching, and sheltering opportunities available to birds and thereby accommodate species with different body sizes, mobility traits, and resource-use strategies [37,38]. A similar distinction was evident among the visual metrics. Visual FDis and visual Rao’s Q responded strongly to tree-related variables, whereas mean visual aesthetic attractiveness, visual CWM, and visual range lacked consistent predictive support. This suggests that vegetation-associated variation in visual aesthetic attractiveness was expressed more consistently in within-community score dispersion than in shifts in community-level mean attractiveness. Greater dispersion may provide opportunities for residents to encounter birds with more diverse levels of visual attractiveness but should not be interpreted as a higher overall aesthetic value of the community.

The major tree-related predictors also showed pronounced nonlinear response patterns for both functional and visual metrics. SHAP responses generally strengthened as tree cover, tree height, or tree richness increased, but subsequently leveled off or declined. This pattern indicates that increases in vegetation structure were not associated with uniformly increasing model responses. Greater vegetation structure may initially enhance resource availability, vertical habitat space, and habitat heterogeneity, whereas additional increases may provide diminishing benefits or alter the balance between open and densely vegetated habitat. Similar nonlinear responses to fine-scale habitat structure have been reported in urban bird communities. For example, Ref. [41] found higher bird diversity at intermediate vegetation heights, highlighting the importance of structural heterogeneity rather than simple increases in vegetation quantity. Nevertheless, the models showed moderate predictive performance (OOF R^2^ = 0.250–0.404), and alternative species-weighting schemes reduced predictive performance relative to the original annual models. Accordingly, the higher-response ranges identified in this study should be interpreted as portions of the sampled environmental gradients associated with relatively strong model responses, rather than as universal ecological thresholds or management targets. The relative importance of tree-related predictors also depended on the observational-weighting scheme. Resident-only models broadly retained the tree-variable rankings of the original annual models, whereas square-root and presence/absence weighting produced more pronounced changes, particularly for visual diversity. For example, total tree cover ranked first for visual FDis under annual weighting but declined to tenth under presence/absence weighting, while tree richness declined from second to thirteenth (Table S10). These results indicate that the apparent importance of some environmental predictors is sensitive to how bird observations are weighted, especially when alternative models have weaker predictive performance.

### 4.2. Interpreting Common Environmental Responses and Local Divergence

Bird functional diversity and visual-attractiveness diversity were positively associated across transects and showed broadly concordant responses to several environmental gradients. In particular, matched FDis and Rao’s Q metrics remained positively correlated after controlling for bird species richness and cumulative observation counts and exhibited concordant nonlinear responses to major tree-related variables. By contrast, the association between functional FRic and visual range was considerably weaker, indicating that correspondence between the functional and visual dimensions was not uniform across metrics. However, FRic measures the extent of a two-dimensional functional space, whereas visual range represents the difference between extreme scores on a single axis. Their weaker association may therefore partly reflect differences in metric construction [30]. Previous studies have likewise reported links between ecological and broader cultural dimensions of bird communities. Urbanization can reduce both functional and cultural richness [20], while ecological and cultural functional groups can remain correlated after accounting for species richness and spatial structure [18]. Our results complement this evidence by examining the dispersion of species-level visual-attractiveness scores, showing that corresponding functional and visual community attributes can exhibit common responses to the same environmental gradients at the scale of urban residential areas.

One plausible explanation for these common responses is the shared dependence of functional and visual metrics on bird community composition. The paired Rao’s Q metrics share a weighted pairwise-distance formulation, while the paired FDis metrics use analogous weighted dispersion measures. Environmental filtering can alter which species occur in a community and, consequently, the distributions of both functional traits and visual attributes represented by those species. Previous studies have shown that landscape structure can influence the distribution of traits associated with bird attractiveness [39], while environmental context can also reshape trait composition associated with regulating and cultural ecosystem services [19]. Thus, similar functional and visual responses may emerge when the same environmental filter reorganizes the species pool underlying both sets of metrics. Importantly, however, this does not necessarily imply an independent synergistic relationship between the two dimensions. The permutation-based null analyses further showed that most observed functional–visual associations and response-curve similarities did not consistently exceed expectations generated by shared community composition. However, the limited discrimination of the four linear curve comparisons means that these null results cannot be interpreted as demonstrating the absence of shared environmental responses. The observed concordance is therefore better interpreted as common environmental responses that may partly reflect shared community composition and metric construction, rather than as evidence of an independent synergistic mechanism.

Despite this overall concordance, localized opposing responses occurred for a small number of shrub- and herb-layer variables. Total herbaceous cover elicited opposing responses in the functional–visual FDis pair, while total shrub cover showed a similar divergence in the Rao’s Q pair. However, the joint relative contributions of these variables were substantially lower than those of the major tree-related variables, and concordant responses remained dominant overall. One plausible explanation is that variation in understory vegetation alters habitat structure and resource availability, thereby favoring species with different ecological traits or habitat requirements. Previous studies have similarly shown that vegetation complexity and resource composition can differentially affect avian functional diversity and ecological groups [42]. At the same time, visual aesthetic attractiveness is shaped by traits such as plumage coloration, body size, and ornamental features, which need not correspond directly to the ecological traits favored under particular habitat conditions [4,43]. Functional and visual responses may therefore diverge locally even when their broader environmental responses remain concordant. Accordingly, these divergent predicted responses are best interpreted as localized departures from an otherwise common response pattern, rather than as demonstrated ecological trade-offs or a general conflict between bird functional diversity and visual-attractiveness diversity.

### 4.3. Vegetation Management Implications of Common and Divergent Environmental Responses

Under the primary annual observational-weighting scheme, the most influential shared predictors of bird functional diversity and visual-attractiveness diversity were total tree cover, tree richness, and mean tree height. Management could therefore consider the retention and improvement of tree structure associated with favorable responses in both dimensions. Existing mature trees could be retained, and unnecessary canopy loss associated with facility upgrades or standardized pruning should be minimized. Research on urban vegetation change has also shown that gains in vegetation area do not necessarily compensate for losses in vegetation quality, further supporting the retention of established vegetation [44]. Where tree structure is limited, increasing tree richness and vertical heterogeneity may improve habitat complexity. Previous studies similarly show that woody plant composition, tree canopy cover, and vegetation structure can strongly influence urban bird diversity and community composition [40,45].

However, management of these high-contribution tree variables should not assume that more vegetation is always better. Their responses were strongly nonlinear, with modeled contributions generally weakening or declining beyond particular portions of the sampled gradients. Management priorities should therefore depend on existing vegetation conditions. In residential areas with sparse tree cover or relatively simple vegetation structure, priority can be given to improving canopy continuity, tree richness, and vertical stratification. In areas where tree canopies are already relatively continuous and structurally developed, further increases in canopy cover or tree height were not necessarily associated with stronger model predictions. Instead, management can emphasize tree-species composition and the maintenance of structural heterogeneity. This interpretation is consistent with evidence that intermediate vegetation height and greater habitat complexity can support urban bird diversity and associated ecosystem services [41]. Accordingly, the higher-response ranges identified in this study should be treated as context-dependent references within the sampled environmental gradients, rather than as universal design thresholds.

Management of shrub and herb layers could also consider localized divergences in predicted functional and visual responses. Although several understory variables showed opposing responses for individual metric pairs, their joint contributions were substantially lower than those of the major tree-related variables. These layers should therefore neither be removed simply because localized response divergence was observed nor increased uniformly in pursuit of a single response. Instead, management should maintain spatial heterogeneity by retaining a mosaic of shrub patches, herbaceous areas, leaf-litter patches, and more open ground while avoiding extensive understory clearing and excessive homogenization. Ground-level vegetation structure and tree-level vegetation have been shown to support different components of urban bird communities, reinforcing the value of maintaining heterogeneous vegetation rather than a uniform structure [38].

Overall, management should be differentiated according to the existing vegetation baseline. Residential areas with sparse vegetation and simple structure should first restore basic habitat components, whereas sites with well-developed tree canopies should place greater emphasis on plant composition and structural heterogeneity. Such a differentiated strategy could help maintain heterogeneous bird habitats while accounting for diversity in morphological functional traits and species-level visual-attractiveness scores. Its effects on bird communities and residents’ experiences would require direct evaluation.

### 4.4. Limitations and Future Directions

This study was conducted in 33 residential areas along the Min River in Fuzhou. Accordingly, the environmental responses and higher-response ranges identified here are most directly applicable to residential environments represented by this river-corridor sample. The 78 transects were nested within only 33 residential areas, and grouped validation does not fully exclude spatial dependence between neighboring residential areas. Future studies should include residential areas across different urban locations, development densities, and climatic regions to evaluate the generality and transferability of the common environmental responses and nonlinear patterns identified here.

This study used standardized species-level scores from iRateBirds to characterize bird visual aesthetic attractiveness. These scores are based on large-scale photographic evaluations and provide a consistent basis for comparisons among bird species, but they do not directly represent place-based evaluations by Fuzhou residents encountering birds in everyday residential settings. Future studies could combine local resident surveys with community-level visual metrics to test whether the environmental-response patterns identified here remain consistent under local, in situ evaluations. Previous studies have combined quantitative landscape descriptors or audio-visual scenes with participants’ reported restorative and emotional responses [46,47], providing methodological references for future assessments of residents’ perceptions of urban birds.

Several additional limitations concern data collection and interpretation. Construction-year information obtained from the commercial real-estate platform may contain reporting inaccuracies. Annual cumulative observations represent relative observational weights rather than detection-corrected abundance estimates, and detectability may vary among species, seasons, and vegetation conditions. Annual aggregation can obscure seasonal variation, while vegetation was measured only once in spring, potentially affecting interpretations of understory responses. Sensitivity analyses indicated that some model results depended on species weighting and functional-trait representation. The relative importance of key tree-related predictors also varied across alternative weighting schemes, limiting the generalizability of their rankings. The conditional XGBoost null tests held previously selected model configurations and response-curve families fixed and therefore did not incorporate uncertainty arising from repeating the full model-selection process. Furthermore, functional and visual indices share species composition, observational weights, and aspects of their mathematical structure. Finally, the observational design and SHAP analyses cannot establish causal effects, directly quantify ecosystem functioning, or identify universal management thresholds. Tree height and first-branch height were visually estimated rather than instrument-measured, potentially introducing additional measurement uncertainty.

## 5. Conclusions

Bird functional-trait diversity and diversity in species-level visual-attractiveness scores showed broadly concordant predicted environmental responses in urban residential areas. Within the sampled Min River corridor, internal vegetation structure provided more consistent predictive information than the surrounding-landscape variables considered, with total tree cover, mean tree height, and tree richness emerging as the principal shared predictors. Several of these responses were strongly nonlinear. Matched FDis and Rao’s Q metrics were positively associated and responded concordantly to major tree-related gradients, whereas local response divergence occurred mainly for a few low-contribution shrub- and herb-layer variables. Because most observed functional–visual associations and response-curve similarities did not consistently exceed expectations generated by shared community composition, these patterns are best interpreted as common environmental responses rather than evidence of an independent synergistic mechanism. These interpretations are conditional on the observational weighting scheme and the moderate predictive performance of the models.

These findings suggest considering vegetation management that prioritizes mature-tree retention, context-appropriate canopy cover, tree richness, and structural heterogeneity while adapting shrub- and herb-layer management to existing site conditions rather than uniformly increasing vegetation cover. By integrating bird functional diversity with community-level visual-attractiveness diversity, this study provides a context-dependent, model-based reference for residential vegetation management, without directly demonstrating improvements in ecosystem functioning or residents’ visual experiences.

## Figures and Tables

**Table 1:** Descriptive statistics of bird functional diversity and visual-attractiveness metrics across 78 transects.

Dimension	Metric	Median	Range
Functional diversity	FRic	12.54	2.51–22.48
	FDis	1.34	0.55–2.26
	Rao’s Q	1.83	0.81–2.96
visual aesthetic attractiveness	Mean visual aesthetic attractiveness	5.96	5.73–6.20
	Visual CWM	5.82	5.46–6.15
	Visual FDis	0.529	0.205–0.650
	Visual Rao’s Q	0.691	0.337–0.821
	Visual range	2.032	1.659–2.794

## Data Availability

The de-identified environmental predictors, derived functional and visual diversity metrics, residential-area grouping identifiers, selected model outputs, and R scripts are provided in the accompanying Supplementary Data and Code archive. The archive also includes the annual observation-count matrix for 78 transects and 56 bird species, their relative observational weights, and corresponding functional traits and visual-attractiveness scores linked by pseudonymous species identifiers (B001–B056). These materials support independent reconstruction of the annual diversity metrics and reproduction of the species-score permutation analyses, fixed-configuration conditional XGBoost null tests, and principal environmental models. Visit-level and seasonal bird observations, precise sampling locations, the species-name mapping, and individual-plant field-survey records are not included because they form part of an ongoing research dataset. Bird functional-trait data were obtained from the publicly available AVONET dataset, and species-level visual aesthetic attractiveness scores were obtained from the publicly available iRateBirds dataset.

## Supplementary Materials

The following supporting information can be downloaded at https://www.mdpi.com/article/10.3390/biology15211972/s1. Supplementary Methods SM1: Grouped Nested Cross-Validation and XGBoost Implementation; SM2: Alternative Observational Weighting and Functional-Trait Sensitivity Analyses; SM3: OOF-SHAP Response Classification and Bootstrap Uncertainty; SM4: Permutation-Based Null-Model Procedures; SM5: Landscape-Buffer Overlap and Spatial Autocorrelation Diagnostics. Table S1: Environmental predictors, spatial scales, and data sources used in the analysis; Table S2: Out-of-fold predictive performance of functional and visual response models; Table S3: PCA loadings of the seven AVONET morphological traits used to construct the two-dimensional functional space; Table S4: Robustness of matched functional–visual responses; Table S5: Sensitivity of model performance and functional–visual associations to alternative observational weighting and community representations; Table S6: Sensitivity of functional diversity metrics to two-dimensional PCA and seven-trait representations; Table S7: Cluster-bootstrap stability of key OOF-SHAP response patterns, turning locations, and predictor rankings; Table S8: Moran’s I of residential-area-aggregated OOF residuals under alternative spatial-neighbor definitions; Table S9: Permutation-based evaluation of functional–visual associations and shared environmental responses; Table S10: Sensitivity of key tree-related predictor rankings to alternative community-weighting schemes. Figure S1: Out-of-fold predictive performance of the functional and visual response models; Figure S2: Landscape-scale selection frequency across outer cross-validation folds; Figure S3: Selected OOF-SHAP responses for Functional FRic; Figure S4: Selected OOF-SHAP responses for Functional FDis; Figure S5: Selected OOF-SHAP responses for Functional Rao’s Q; Figure S6: Selected OOF-SHAP responses for Visual FDis; Figure S7: Selected OOF-SHAP responses for Visual Rao’s Q; Figure S8: Functional–visual associations across all metric pairs; Figure S9: Functional–visual common environmental responses and local response divergence across environmental predictors; Figure S10: Shared-community-composition null distributions for the matched FDis and Rao’s Q associations based on 4999 frequency-constrained permutations of species-level visual scores; Figure S11: Robustness of functional–visual response classification to alternative rule settings (a) and leave-one-residential-area-out analysis (b); Figure S12: Sensitivity of OOF-SHAP response curves to alternative species-weighting schemes. References [48,49,50,51,52] are cited in the Supplementary Materials.
