# Supplementary material for: Common Environmental Responses of Bird Functional and Visual-Attractiveness Diversity in Urban Residential Areas
Source: Biology (Basel). 2026 Nov 2;15(21):1972. doi: 10.3390/biology15211972 (PMC13761421; doi:10.3390/biology15211972)

Supplementary Materials for:

Common Environmental Responses of Bird Functional and Visual-Attractiveness Diversity in Urban Residential Areas

Authors: Deng Pan[a], Yaqin Ding[a], Yujun Chen[a], Siteng Wu[a], Ziang Su[a], Juan Wang[a], Zhipeng Zhu[b], Yuxiang Liu[c], Xiaoling Xu[a], Weicong Fu[a]*

Affiliations:

[a] College of Landscape Architecture and Art, Fujian Agriculture and Forestry University, Fuzhou 350002, China

[b] College of Architecture and Urban Planning, Fujian University of Technology, Fuzhou 350118, China

[c] Faculty of Forestry & Environmental Stewardship, University of British Columbia, Vancouver, BC, V6T 1Z4, Canada

ORCID: Deng Pan, 0009-0006-0690-0319

Corresponding author: Weicong Fu

Email address: weicongfufj@163.com

Full postal address: College of Landscape Architecture and Art, Fujian Agriculture and Forestry University, Fuzhou 350002, China

SM1. Grouped Nested Cross-Validation and XGBoost Implementation

The 78 transects were nested within 33 residential areas. Residential-area identifiers were used as grouping variables throughout model validation to prevent transects from the same residential area from appearing simultaneously in training and testing sets. This approach follows recommendations for cross-validation of spatially and hierarchically structured ecological data [1].

We employed repeated nested cross-validation with five outer folds repeated three times and four inner folds. Candidate models included internal environmental variables alone or additional landscape predictors measured at 300, 500, 1,000, or 1,500 m. Predictor screening and model selection were performed using training data without access to the corresponding outer testing responses. Within training folds, predictors were assessed for missingness, the number of distinct values, and pairwise Spearman correlations. The screening criteria included a maximum missing-data proportion of 20%, at least four distinct values, and an absolute correlation threshold of 0.85. When correlated predictors were screened, priority was given to internal vegetation variables, followed by built-environment and external landscape variables.

Spatial-scale selection and hyperparameter optimization were conducted within the inner cross-validation procedure. Candidate configurations were evaluated using RMSE and a one-standard-error selection rule favoring simpler models. Hyperparameter optimization considered 12 configurations, comprising one baseline configuration and 11 seeded selections from the candidate parameter grid. The parameters included tree depth, learning rate, minimum child weight, row and column subsampling, and regularization terms. Models used squared-error regression, histogram-based tree construction, and early stopping with a maximum of 1,400 boosting rounds and 60 rounds without improvement [2]. The complete candidate parameter settings and random-seed configuration are retained in the analysis scripts.

After model selection, the selected configuration was refitted using the outer training data, with the number of boosting iterations determined without using outer testing observations. Each transect received three OOF predictions across the repeated outer validation runs. These predictions were averaged before calculating OOF R^2, RMSE, MAE, and Spearman correlations.

The original input preparation included a preliminary full-data quality check. All 38 candidate predictors passed this check, and no predictor was removed through that global screening step. Subsequent predictor screening and model selection were conducted within the training folds. Therefore, the preliminary check did not alter the candidate predictor set in the analyzed dataset, although this distinction is retained for transparency and reproducibility.

SM2. Alternative Observational Weighting and Functional-Trait Sensitivity Analyses

The original annual community matrix contained cumulative species observation counts from 78 transects and 56 bird species. For each transect, species counts were normalized to obtain relative observational weights for calculating functional and visual diversity metrics. These weights describe the relative composition of recorded observations and do not constitute detection-corrected estimates of species abundance.

To assess sensitivity to the weighting scheme, we recalculated the four core diversity metrics using square-root-transformed counts and presence/absence weights. Square-root weighting was obtained by transforming annual species counts before normalizing them to sum to one within each transect. Under presence/absence weighting, species recorded in a transect received equal relative weights. The square-root transformation was used to reduce the influence of frequently recorded species, consistent with established community-data transformation approaches [3].

We additionally verified the standard Hellinger transformation. When the resulting Hellinger values were further normalized to sum to one within each transect, the normalization factor canceled, producing weights mathematically identical to those obtained from square-root-transformed counts. Accordingly, Hellinger-based normalized weights were not treated as an independent sensitivity analysis.

We also recalculated community metrics for resident-only bird assemblages and separately for spring, summer, autumn, and winter. The resident-only subset contained 40 species. These analyses examined whether annual community aggregation and seasonal species occurrence affected the observed functional–visual associations and environmental model performance. They do not correct differences in detection probability or establish that each recorded observation represents a distinct individual.

Finally, functional FDis and Rao's Q were recalculated using Euclidean distances in the complete seven-trait standardized morphological space, rather than the two-dimensional PCA space. Six positive morphological measurements were log10-transformed, whereas the hand-wing index remained untransformed; all seven traits were subsequently z-score standardized. For both trait representations, FDis was calculated as the observationally weighted dispersion around the community centroid, and Rao's Q was calculated from weighted pairwise Euclidean trait distances [4,5]. The alternative metrics were compared with the original two-PC metrics, and the corresponding XGBoost models were reevaluated using the same grouped nested cross-validation design.

Detailed comparisons of model performance and functional–visual associations are provided in Table S5, while the effects of trait-space representation are summarized in Table S6. To further examine the sensitivity of predictor importance to observational weighting, we compared the ranks of total tree cover, mean tree height, and tree richness across the original annual, square-root, presence/absence, and resident-only models. Predictor ranks were based on mean absolute OOF-SHAP values within each model, with results summarized in Table S10.

SM3. OOF-SHAP Response Classification and Bootstrap Uncertainty

SHAP values were calculated for observations in the outer testing folds and used to characterize the contributions of environmental predictors to fitted model predictions [6,7]. Predictor importance was summarized using mean absolute OOF-SHAP values. The dependence plots displayed transect-level SHAP contributions and smoothed environmental response patterns.

Three complementary residential-area cluster-bootstrap analyses were used to assess different aspects of interpretation uncertainty, following the general principles of resampling clustered observations [8].

First, 400 residential-area cluster-bootstrap resamples were used to estimate uncertainty in binned median OOF-SHAP contributions. The resulting 95% percentile intervals are displayed as error bars around the binned medians in Figure 4. The shaded bands around the LOESS-smoothed curves represent approximate pointwise intervals from the smoothing procedure and are distinct from the cluster-bootstrap intervals. Neither approach involves refitting the complete XGBoost pipeline or accounts for uncertainty associated with model selection.

Second, response-shape stability was assessed using 250 residential-area cluster-bootstrap resamples of the existing OOF-SHAP data. The selected linear or natural-spline response representation was refitted to each resampled dataset. Response classes included positive, negative, hump-shaped, U-shaped, and more complex or weakly supported patterns. Shape support was quantified as the proportion of resamples retaining the original response classification. Responses were considered stable when the original shape was retained in at least 70% of resamples and the median bootstrap curve correlation was

at least 0.70. For stable curves with identifiable internal extrema, turning locations were summarized by their median and the 2.5th and 97.5th percentiles of finite bootstrap estimates.

Third, predictor-ranking stability was assessed using 1,000 residential-area cluster-bootstrap resamples of the original OOF-SHAP contributions. Predictor ranks were recalculated for each resample, and their median ranks, percentile intervals, and frequencies of appearing among the three or five highest-ranking predictors were summarized.

These analyses evaluate uncertainty conditional on the previously fitted models and selected analytical representations. They do not account for all uncertainty associated with repeating model selection, hyperparameter optimization, or data collection. Detailed results for key predictors are reported in Table S7.

Matched functional and visual response curves were further compared using curve correlations, concordant and divergent response intervals, and joint relative contributions. Classification robustness was assessed under 108 alternative rule settings and through leave-one-residential-area-out analyses. The corresponding results are summarized in Table S4 and Figure S11. These comparisons characterize model-predicted response agreement and divergence rather than demonstrating causal synergy or ecological trade-offs.

SM4. Permutation-Based Null-Model Procedures

Two permutation-based null analyses were conducted to evaluate whether functional–visual associations and similarities in environmental response patterns could arise from the shared species composition of bird communities. The permutation design followed the general principles of constrained ecological null models and species-attribute randomization [9,10].

For the species-score permutation analysis, the original annual transect-by-species observation matrix and functional-trait coordinates were held constant. Only the correspondence between bird species and their visual-attractiveness scores was randomized. Species occupancy frequency was calculated as the number of the 78 transects in which each species occurred. The 56 species were ranked by occupancy frequency and divided into four equal-sized strata containing 14 species each. Within each stratum, visual-attractiveness scores were randomly reassigned among species without replacement. Species with identical occupancy frequencies could fall into different strata because ties were resolved by their original ordering. Consequently, this procedure represented approximate occupancy-frequency-stratified permutation rather than an exact matching of species with identical occupancy frequencies.

A total of 4,999 constrained permutations were performed. We also conducted 4,999 unrestricted permutations in which visual-attractiveness scores were randomly reassigned across all 56 species. For every permutation, visual FDis and visual Rao's Q were recalculated while preserving the original community observations and functional metrics. Raw Spearman correlations and partial Spearman correlations controlling for bird species richness and cumulative bird observations were then recalculated for the matched functional–visual metric pairs.

The conditional XGBoost null analysis used 199 occupancy-frequency-constrained permutations. For each permutation, visual-attractiveness scores and the corresponding visual diversity metrics were recalculated. Visual XGBoost models were then refitted using the original outer training groups, and OOF-SHAP values were recomputed for the corresponding outer testing groups. The previously selected spatial scales, predictors, hyperparameters, and boosting-iteration counts were held fixed. Functional-model SHAP results and the selected functional reference curves were held constant. Each observed and permuted visual target used its originally selected linear or three-df natural-spline representation on a

shared 121-point predictor grid. The resulting null distributions were used to evaluate matched functional–visual response-curve correlations and joint relative predictor contributions.

The full inner cross-validation procedure, spatial-scale selection, hyperparameter optimization, and visual-curve family selection were not repeated for every conditional permutation. Therefore, the conditional null analysis tested the consequences of species-score reassignment under fixed, previously selected model configurations. It did not represent an unconditional null distribution for the entire model-selection pipeline.

Empirical upper-tail and absolute-tail P values were calculated using the observed statistic and the corresponding number of randomized statistics meeting the specified tail criterion, with one added to both the numerator and denominator [11]. For the absolute-tail test, permutation statistics were compared using their absolute magnitudes rather than deviations from the potentially nonzero null-distribution mean. The reported 95% null intervals were obtained from the 2.5th and 97.5th percentiles of the randomized statistics. Accordingly, absolute-tail P values and equal-tailed null intervals do not necessarily yield identical threshold decisions.

The smallest attainable empirical P values were 0.0002 for 4,999 permutations and 0.005 for 199 permutations. The randomization analyses used the recorded base seed of 20260828, with additional derived seeds used for conditional model fitting. Null-model results are summarized in Tables S4 and S9 and Figure S10. These tests were designed to assess whether the observed concordance exceeded randomization expectations under the specified constraints, not to establish or exclude causal relationships between functional and visual attributes.

SM5. Landscape-Buffer Overlap and Spatial Autocorrelation Diagnostics

Surrounding-landscape variables were calculated within 300-, 500-, 1,000-, and 1,500-m buffers around the 33 residential-area analysis polygons used for GIS extraction. The corresponding residential-area polygons were excluded when calculating surrounding-landscape variables.

To assess spatial overlap, we calculated the summed area of individual buffers and the area of their geometric union for each spatial scale. The overlapping-area redundancy fraction was defined as one minus the union area divided by the summed area of the individual buffers. For the external buffer areas excluding each residential-area polygon, the redundancy fractions were 2.9%, 10.1%, 40.5%, and 58.1% at 300, 500, 1,000, and 1,500 m, respectively. These values describe the proportion of duplicated buffer area rather than the average pairwise percentage overlap. Increasing overlap at larger spatial scales may contribute to similarity among landscape predictors and limit the independence of surrounding-landscape information.

Residual spatial autocorrelation was evaluated using OOF prediction residuals aggregated at the residential-area level. For each transect, the three repeated OOF predictions were averaged, and the residual was calculated as the observed response minus the averaged prediction. Transect residuals were then averaged within each of the 33 residential areas.

Spatial weights were constructed from residential-area polygon centroids using symmetrized four-nearest-neighbor and six-nearest-neighbor graphs, followed by row standardization. Moran's I was calculated for each of the five retained response models. Empirical P values were obtained from 4,999 spatial permutations by comparing the absolute deviation of the observed Moran's I from its expected value under spatial randomness with the corresponding permutation deviations. Benjamini–Hochberg correction was applied separately across the five response models under each neighbor definition.

No statistically significant residual spatial autocorrelation was detected under either neighborhood specification after multiple-testing correction (Table S8). Nevertheless, these diagnostics do not establish

complete spatial independence or eliminate potential dependence at other spatial scales. The overlapping landscape buffers and hierarchical sampling structure should therefore be considered when interpreting model predictions and their transferability.

References for Supplementary Materials

1. Roberts, D.R.; Bahn, V.; Ciuti, S.; Boyce, M.S.; Elith, J.; Guillera-Arroita, G.; Hauenstein, S.; Lahoz-Monfort, J.J.; Schröder, B.; Thuiller, W.; et al. Cross-validation strategies for data with temporal, spatial, hierarchical, or phylogenetic structure. Ecography 2017, 40, 913–929. https://doi.org/10.1111/ecog.02881

2. Chen, T.; Guestrin, C. XGBoost: A Scalable Tree Boosting System. In Proceedings of the 22nd ACM SIGKDD International Conference on Knowledge Discovery and Data Mining, San Francisco, CA, USA, 13–17 August 2016; ACM: New York, NY, USA, 2016; pp. 785–794. https://doi.org/10.1145/2939672.2939785

3. Legendre, P.; Gallagher, E.D. Ecologically meaningful transformations for ordination of species data. Oecologia 2001, 129, 271–280. https://doi.org/10.1007/s004420100716

4. Laliberté, E.; Legendre, P. A distance-based framework for measuring functional diversity from multiple traits. Ecology 2010, 91, 299–305. https://doi.org/10.1890/08-2244.1

5. Botta-Dukát, Z. Rao's quadratic entropy as a measure of functional diversity based on multiple traits. Journal of Vegetation Science 2005, 16, 533–540. https://doi.org/10.1111/j.1654-1103.2005.tb02393.x

6. Lundberg, S.M.; Lee, S.-I. A Unified Approach to Interpreting Model Predictions. In Advances in Neural Information Processing Systems; Curran Associates, Inc.: Red Hook, NY, USA, 2017; Volume 30. Available online: https://proceedings.neurips.cc/paper_files/paper/2017/hash/8a20a8621978632d76c43dfd28b67767-Abstract.html (accessed on 30 October 2026).

7. Lundberg, S.M.; Erion, G.; Chen, H.; DeGrave, A.; Prutkin, J.M.; Nair, B.; Katz, R.; Himmelfarb, J.; Bansal, N.; Lee, S.-I. From local explanations to global understanding with explainable AI for trees. Nature Machine Intelligence 2020, 2, 56–67. https://doi.org/10.1038/s42256-019-0138-9

8. Field, C.A.; Welsh, A.H. Bootstrapping clustered data. Journal of the Royal Statistical Society: Series B (Statistical Methodology) 2007, 69, 369–390. https://doi.org/10.1111/j.1467-9868.2007.00593.x

9. Gotelli, N.J. Null model analysis of species co-occurrence patterns. Ecology 2000, 81, 2606–2621. https://doi.org/10.1890/0012-9658(2000)081[2606:NMAOSC]2.0.CO;2

10. Dray, S.; Legendre, P. Testing the species traits–environment relationships: The fourth-corner problem revisited. Ecology 2008, 89, 3400–3412. https://doi.org/10.1890/08-0349.1

11. Phipson, B.; Smyth, G.K. Permutation P-values should never be zero: Calculating exact P-values when permutations are randomly drawn. Statistical Applications in Genetics and Molecular Biology 2010, 9, Article 39. https://doi.org/10.2202/1544-6115.1585

Table S1. Environmental predictors, spatial scales, and data sources used in the analysis.

Category	Predictor	Scale	Data source
**Internal vegetation**	Mean tree height	Transect	Field vegetation survey
	Total tree / shrub / herbaceous cover	Transect	Field vegetation survey
	Tree / shrub / herbaceous richness	Transect	Field vegetation survey
**Built environment**	Green rate	Residential area	Residential spatial data / GIS processing
	Floor area ratio	Residential area	Urban Data Analyst Platform / GIS processing
	Mean building height, and building density	Residential area	Urban Data Analyst Platform / GIS processing
	Residential area size and distance to city center	Residential area	Baidu Maps Open Platform / residential background data
	Building year	Residential area	58 Anjuke (Fuzhou)
**External landscape**	Building, tree, and water cover proportions	300, 500, 1,000, and 1,500 m	SinoLC-1 (1 m)
	Mean NDVI and NDVI SD	300, 500, 1,000, and 1,500 m	National Ecosystem Science Data Center (30 m NDVI)
	Road density	300, 500, 1,000, and 1,500 m	OpenStreetMap / Geofabrik / GIS processing

Note: The 38 candidate predictors comprised seven internal vegetation variables, seven built-environment variables, and 24 surrounding-landscape variables. External landscape predictors were calculated at four spatial scales (300, 500, 1,000, and 1,500 m) using residential-area analysis polygons. Not all candidate predictors were included simultaneously in each fitted model.

Table S2. Out-of-fold predictive performance of functional and visual response models.

Response	OOF R^2 (95% CI)	Spearman ρ	RMSE	MAE
Functional FRic	0.250 (0.042 to 0.392)	0.556	3.803	3.227
Functional FDis	0.364 (0.166 to 0.504)	0.612	0.223	0.155
Functional Rao's Q	0.309 (0.085 to 0.418)	0.534	0.295	0.218
Mean visual aesthetic attractiveness	-0.060 (-0.163 to -0.027)	-0.207	0.094	0.076
Visual CWM	-0.069 (-0.189 to -0.029)	-0.267	0.131	0.100
Visual range	-0.105 (-0.267 to -0.051)	-0.184	0.292	0.215
Visual FDis	0.375 (0.173 to 0.490)	0.629	0.087	0.068
Visual Rao's Q	0.404 (0.154 to 0.520)	0.557	0.092	0.073

Note: OOF predictions were obtained using repeated nested cross-validation grouped by residential area. The 95% confidence intervals for OOF R^2 were estimated from 1,000 residential-area cluster-bootstrap resamples. All performance metrics were evaluated at the transect level, while the 33 residential areas were treated as grouping units.

Table S3. PCA loadings of the seven AVONET morphological traits used to construct the two-dimensional functional space.

Trait	Transformation	PC1 loading	PC2 loading
Bill length (culmen)	log10, then Z-standardized	0.419	-0.105
Bill width	log10, then Z-standardized	0.417	-0.034
Bill depth	log10, then Z-standardized	0.421	0.090
Tarsus length	log10, then Z-standardized	0.393	0.191
Hand-wing index	Untransformed, then Z-standardized	0.083	-0.948
Tail length	log10, then Z-standardized	0.342	0.189
Body mass	log10, then Z-standardized	0.441	-0.093

Note: PC1 and PC2 explained 67.48% and 15.08% of the total trait variance, respectively, accounting for 82.56% cumulatively. PC1 primarily represented overall morphological size, whereas PC2 was predominantly associated with the hand-wing index. These two components were used to construct the functional trait space.

Table S4. Robustness of matched functional–visual responses.

Pair	Predictor	Rule retention (%)	Leave-one retention (%)	Curve ρ obs [95% null interval]	Joint relative contribution [95% null interval]
FDis	Total tree cover	100%	100%	0.97 [-0.98, 1.00]	1.00 [0.33, 1.00]
FDis	Tree richness	58%	100%	1.00 [-1.00, 1.00]	0.44 [0.09, 0.50]
FDis	Mean tree height	50%	100%	0.93 [-0.93, 0.93]	0.31 [0.22, 0.79]
FDis	Building year	50%	97%	0.79 [-0.79, 0.79]	0.15 [0.06, 0.39]
FDis	Total herbaceous cover	25%	100%	-0.87 [-1.00, 1.00]	0.11 [0.06, 0.32]
Rao's Q	Total Tree cover	100%	100%	0.98 [-0.96, 0.99]	0.94 [0.31, 0.94]
Rao's Q	Mean tree height	50%	100%	0.74 [-0.74, 0.74]	0.66 [0.40, 1.00]
Rao's Q	Total shrub cover	100%	100%	-1.00 [-1.00, 1.00]	0.11 [0.13, 0.57]

Note. Rule retention is the percentage of 108 alternative classification-rule settings retaining the baseline response class; leave-one-residential-area-out retention is the percentage of 33 corresponding runs retaining that class. Observed statistics accompany 95% reference null intervals from 199 conditional XGBoost permutations, not confidence intervals for observed statistics. Previously selected model configurations were held fixed without full nested tuning. For four linear targets, identical observed and randomized absolute curve correlations limited discrimination; absolute-tail P = 1 does not demonstrate absence of shared response.

Table S5. Sensitivity of model performance and functional–visual associations to alternative observational weighting and community representations.

(a) Out-of-fold predictive performance (OOF R^2)

Scheme	Functional FDis	Functional Rao's Q	Visual FDis	Visual Rao's Q
Annual	0.364	0.309	0.375	0.404
Square-root	0.175	0.137	0.088	0.085
Presence/absence	0.073	0.007	-0.057	-0.125
Resident-only	0.383	0.354	0.386	0.408
Spring	0.042	0.029	-0.006	0.059
Summer	0.162	0.126	0.095	0.165
Autumn	0.104	0.114	0.170	0.263
Winter	-0.007	-0.078	0.101	0.053

(b) Functional–visual associations (Spearman ρ)

Scheme	FDis raw	FDis partial	Rao's Q raw	Rao's Q partial
Annual	0.627	0.643	0.632	0.612
Square-root	0.427	0.460	0.485	0.487
Presence/absence	0.217	0.280	0.245	0.278
Resident-only	0.668	0.704	0.692	0.685
Spring	0.646	0.708	0.656	0.671
Summer	0.751	0.734	0.707	0.654
Autumn	0.720	0.687	0.695	0.596
Winter	0.674	0.649	0.714	0.679

Note: Annual represents the original within-transect relative observational weighting scheme. Square-root weighting was calculated by applying a square-root transformation to annual species counts before normalization. Presence/absence weighting assigned equal relative weights to species present within each transect. Resident-only and seasonal analyses used the corresponding subsets of bird observations. All environmental models were evaluated using the same residential-area-grouped nested cross-validation framework. Partial Spearman correlations controlled for species richness and cumulative observation counts corresponding to each community subset; the square-root and presence/absence schemes retained the original annual count-based control variables. Correlation uncertainty was additionally assessed using 1,000 residential-area cluster-bootstrap resamples. Standard Hellinger-transformed values, when subsequently normalized to sum to one, yielded weights identical to the square-root scheme and were therefore not treated as an independent sensitivity test.

Table S6. Sensitivity of functional diversity metrics to two-dimensional PCA and seven-trait representations.

Metric	Two-PC OOF R^2	Seven-trait OOF R^2	Spearman ρ between metrics	Median relative change (%)
Functional FDis	0.364	0.293	0.885	+24.5
Functional Rao's Q	0.309	0.383	0.927	+20.6

Note: The original functional diversity metrics were calculated in the two-dimensional PCA trait space, whereas the alternative metrics were calculated using all seven standardized morphological traits. Spearman correlations compare the corresponding transect-level metric values between the two representations. Median relative change was calculated relative to the original two-PC metric values. OOF R^2 values were obtained using the same residential-area-grouped nested cross-validation framework. Differences between the two representations reflect sensitivity to trait-space construction rather than independent validation of ecological functions.

Table S7. Cluster-bootstrap stability of key OOF-SHAP response patterns, turning locations, and predictor rankings.

Response	Predictor	Response shape	Shape support (%)	Turning location, median [95% interval]	Rank median [95% interval]	Top-5 frequency (%)
Functional FRic	Mean tree height	Hump	98.8	11.67 [10.47, 12.13] m	1 [1, 1]	100.0
	Residential area size	Hump	81.2	10.24 [8.43, 11.24] ha	2 [2, 2]	100.0
Functional FDis	Total tree cover	Hump	100.0	64.34 [58.83, 67.32]%	1 [1, 1]	100.0
	Mean tree height	Hump	82.0	13.43 [12.60, 14.16] m	2 [2, 2]	100.0
	Tree richness	Hump	86.4	12.29 [11.42, 12.75]	5 [3, 5]	100.0
	Total herbaceous cover	Positive	98.8	—	9 [8, 10]	0.0
Functional Rao's Q	Total tree cover	Hump	92.0	63.22 [57.63, 68.54]%	2 [1, 2]	100.0
	Mean tree height	Hump	100.0	12.18 [11.79, 12.69] m	1 [1, 2]	100.0
	Tree richness	Hump, unstable	37.2	—	5 [5, 6]	96.1
	Total shrub cover	Positive	100.0	—	4 [4, 4]	100.0
Visual FDis	Total tree cover	Hump	99.6	66.95 [63.59, 68.81]%	1 [1, 1]	100.0
	Tree richness	Hump	84.8	12.00 [11.17, 12.58]	2 [2, 2]	100.0
	Mean tree height	Positive	100.0	—	4 [4, 6]	96.6
	Total herbaceous cover	Hump	98.0	29.62 [27.58, 32.64]%	5 [4, 6]	95.3
Visual Rao's Q	Total tree cover	Hump	100.0	65.46 [60.98, 66.95]%	1 [1, 1]	100.0
	Tree richness	Hump	96.0	11.92 [11.00, 12.58]	2 [2, 2]	100.0
	Mean tree height	Positive	100.0	—	3 [3, 4]	100.0
	Total shrub cover	Negative	99.6	—	11 [10, 13]	0.0

Note: Response-shape stability was assessed using 250 residential-area cluster-bootstrap resamples of

the existing OOF-SHAP data. Shape support denotes the proportion of resamples reproducing the original response class. A response was classified as stable when its shape-support frequency was at least 70% and its median bootstrap curve correlation with the original curve was at least 0.70. Turning locations are reported only for stable responses with identifiable internal extrema. Intervals are the 2.5th and 97.5th percentiles of resamples yielding finite turning locations. Predictor-ranking stability was evaluated using 1,000 residential-area cluster-bootstrap resamples of the original OOF-SHAP contributions. Rank intervals and top-five frequencies are conditional on the fitted models and do not incorporate uncertainty from repeating the entire model-selection procedure. Turning locations should not be interpreted as ecological thresholds.

Table S8. Moran's I of residential-area-aggregated OOF residuals under alternative spatial-neighbor definitions.

**Response**	**4-neighbor Moran's I**	**4-neighbor P**	**4-neighbor BH q**	**6-neighbor Moran's I**	**6-neighbor P**	**6-neighbor BH q**
Functional FRic	0.045	0.4498	0.9113	-0.053	0.7878	0.7878
Functional FDis	-0.024	0.9420	0.9420	-0.114	0.2836	0.6750
Functional Rao's Q	0.030	0.5468	0.9113	-0.098	0.4050	0.6750
Visual FDis	-0.122	0.3758	0.9113	-0.114	0.2928	0.6750
Visual Rao's Q	-0.016	0.8762	0.9420	-0.072	0.6124	0.7655

Note: Transect-level OOF residuals were aggregated to the 33 residential areas before calculating Moran's I. Spatial weights were constructed using symmetrized four- and six-nearest-neighbor graphs based on residential-area centroids, followed by row standardization. Permutation P values were obtained from 4,999 random permutations, using deviations from the expected Moran's I under spatial randomness. Benjamini–Hochberg-adjusted q values were calculated separately across the five response metrics for each neighbor definition. No statistically significant residual spatial autocorrelation was detected under either neighbor definition. However, nonsignificant results do not establish spatial independence or eliminate the possibility of dependence at unexamined spatial scales.

Table S9. Permutation-based evaluation of functional–visual associations and shared environmental responses.

(a) Species-score permutation tests, 4,999 iterations

Null model	Pair	Statistic	Observed ρ	95% null interval	Absolute-tail P	Upper-tail P
Frequency-constrained	FDis	Raw	0.6268	[-0.6279, 0.7158]	0.1286	0.1032
Frequency-constrained	FDis	Partial	0.6430	[-0.6373, 0.6951]	0.0806	0.0572
Frequency-constrained	Rao's Q	Raw	0.6317	[-0.2687, 0.6926]	0.0756	0.0756
Frequency-constrained	Rao's Q	Partial	0.6115	[-0.3424, 0.6270]	0.0322	0.0322
Unrestricted	FDis	Raw	0.6268	[-0.7089, 0.6895]	0.1254	0.0708
Unrestricted	FDis	Partial	0.6430	[-0.6839, 0.6741]	0.0884	0.0442
Unrestricted	Rao's Q	Raw	0.6317	[-0.4186, 0.6662]	0.0518	0.0518
Unrestricted	Rao's Q	Partial	0.6115	[-0.4175, 0.6030]	0.0212	0.0212

(b) Conditional XGBoost permutation tests, 199 iterations

Pair	Predictor	Absolute-tail P for curve ρ	Upper-tail P for curve ρ	Upper-tail P for joint contribution
FDis	Total tree cover	0.245	0.150	0.465
FDis	Tree richness	0.185	0.165	0.085
FDis	Mean tree height	1.000	0.750	0.805
FDis	Building year	1.000	0.435	0.560
FDis	Total herbaceous cover	0.860	0.745	0.785
Rao's Q	Total tree cover	0.050	0.050	0.345
Rao's Q	Mean tree height	1.000	0.920	0.710
Rao's Q	Total shrub cover	1.000	1.000	0.995

Note: Species-level visual-attractiveness scores were permuted while preserving the original bird community matrix and functional traits. Frequency-constrained permutations reassigned scores within four occupancy-frequency strata, whereas unrestricted permutations reassigned scores across all 56 species. Partial Spearman correlations controlled for species richness and cumulative bird observations. Conditional XGBoost permutations refitted visual-response models with selected configurations fixed.

Absolute-tail P = (1 + the number of permutations with absolute statistics at least as large as the observed absolute statistic)/(B + 1). Upper-tail P values compared permutation statistics greater than or equal to the observed statistic. Equal-tailed 95% empirical null intervals and absolute-tail tests use different definitions and need not yield identical decisions. Table S4 reports statistics and intervals. For four linear targets, identical observed and randomized absolute curve correlations limited discrimination; absolute-tail P = 1 does not demonstrate absence of shared response.

Table S10. Sensitivity of key tree-related predictor rankings to alternative community-weighting schemes.

Response	Predictor	Annual	Square-root	Presence/ absence	Resident-only
Functional FDis	Tree cover	1	3	5	1
	Mean tree height	2	2	2	2
	Tree richness	5	5	7	4
Functional Rao’s Q	Tree cover	2	4	8	1
	Mean tree height	1	2	3	2
	Tree richness	5	7	7	5
Visual FDis	Tree cover	1	3	10	1
	Mean tree height	4	11	7	4
	Tree richness	2	2	13	2
Visual Rao’s Q	Tree cover	1	3	6	1
	Mean tree height	3	7	8	3
	Tree richness	2	1	12	2

Note: Predictor ranks were determined from mean absolute OOF-SHAP values within each response model. Lower ranks indicate greater relative importance. Annual, square-root, presence/absence, and resident-only refer to the community-weighting schemes described in Supplementary Methods SM2. Rankings are descriptive and should be interpreted alongside model predictive performance (Table S5), particularly when alternative models showed weak or negative OOF R^2. They do not indicate statistical significance or causal effects.

Figure S1. Out-of-fold predictive performance of the functional and visual response models. Points show OOF R^2 and horizontal lines show 95% cluster-bootstrap confidence intervals.

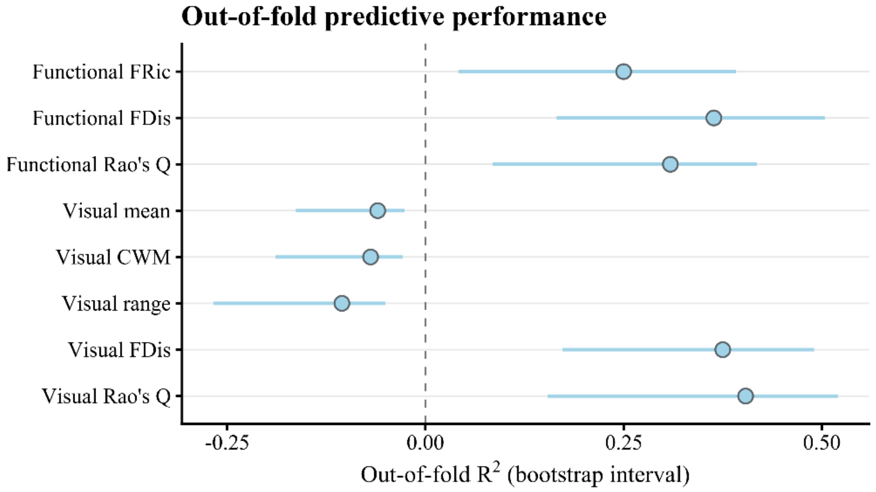

Figure S2. Landscape-scale selection frequency across outer cross-validation folds. "Local only" denotes models without external landscape predictors.

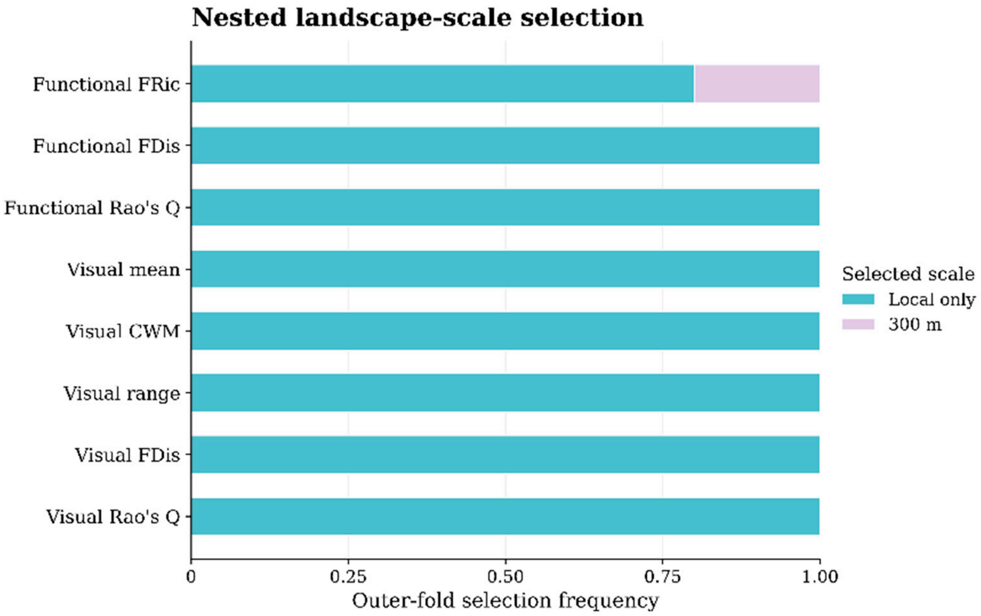

Figure S3. Selected OOF-SHAP responses for Functional FRic. Points show OOF-SHAP values; solid curves show LOESS-smoothed trends, and shaded bands represent smoothed 95% residential-area cluster-bootstrap intervals for binned median SHAP values; horizontal dashed lines indicate SHAP = 0; vertical dotted lines indicate candidate response turning locations.

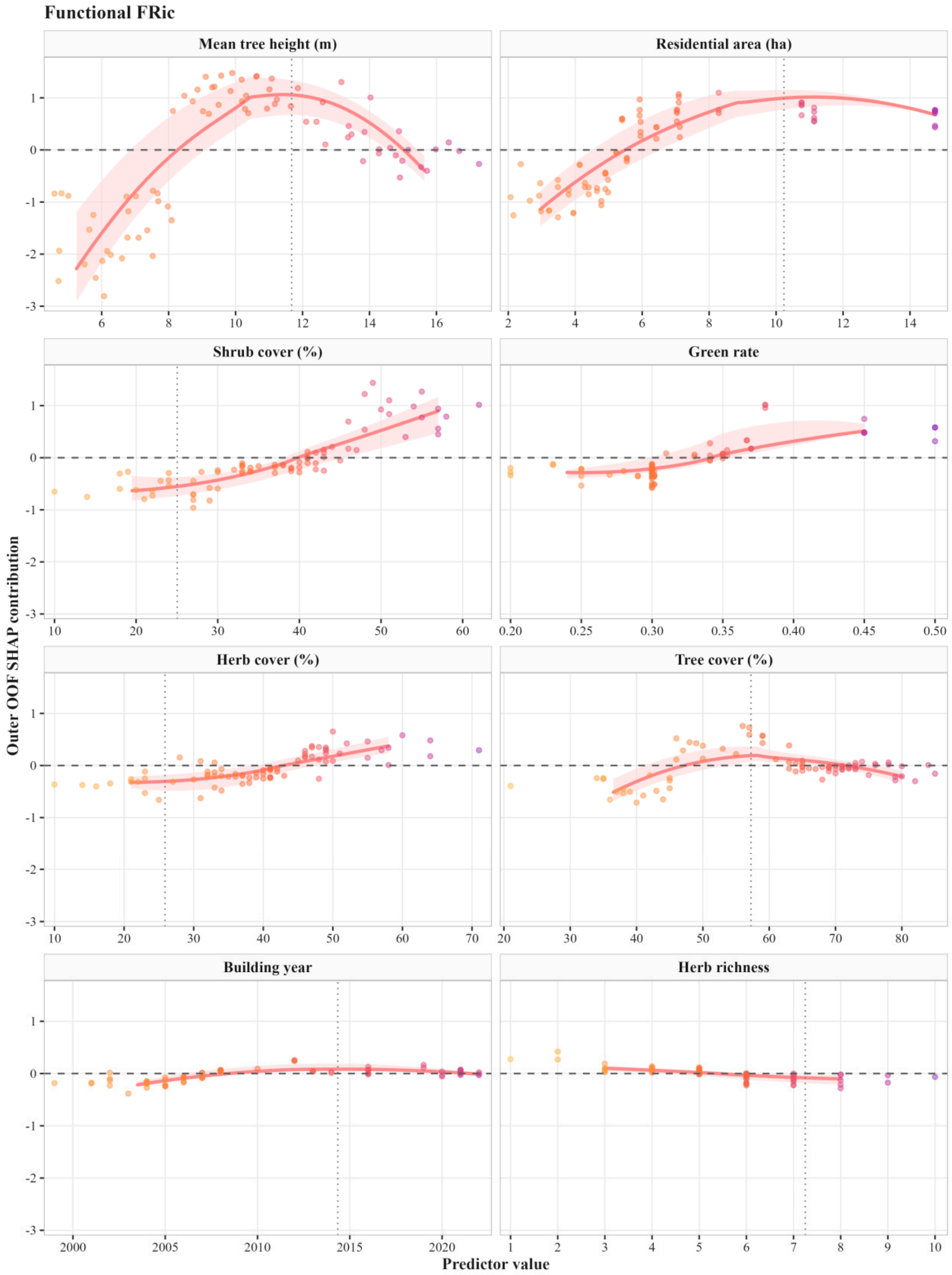

Figure S4. Selected OOF-SHAP responses for Functional FDis. Points show OOF-SHAP values; solid curves show LOESS-smoothed trends; shaded bands indicate smoothed 95% residential-area cluster-bootstrap intervals for binned median SHAP values; horizontal dashed lines indicate SHAP = 0; vertical dotted lines indicate candidate response turning locations.

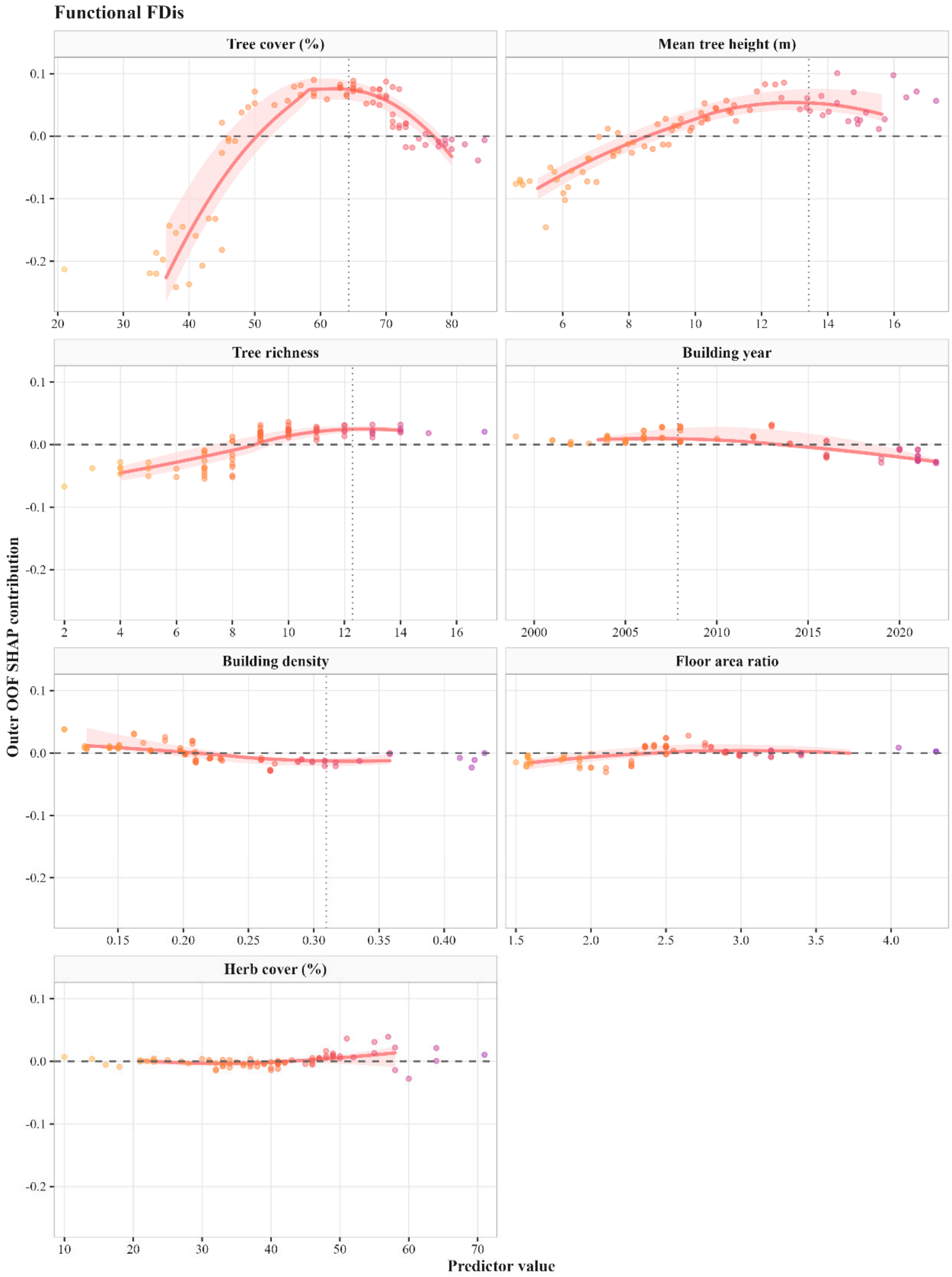

Figure S5. Selected OOF-SHAP responses for Functional Rao's Q. Points show OOF-SHAP values; solid curves show LOESS-smoothed trends; shaded bands indicate smoothed 95% residential-area cluster-bootstrap intervals for binned median SHAP values; horizontal dashed lines indicate SHAP = 0; vertical dotted lines indicate candidate response turning locations.

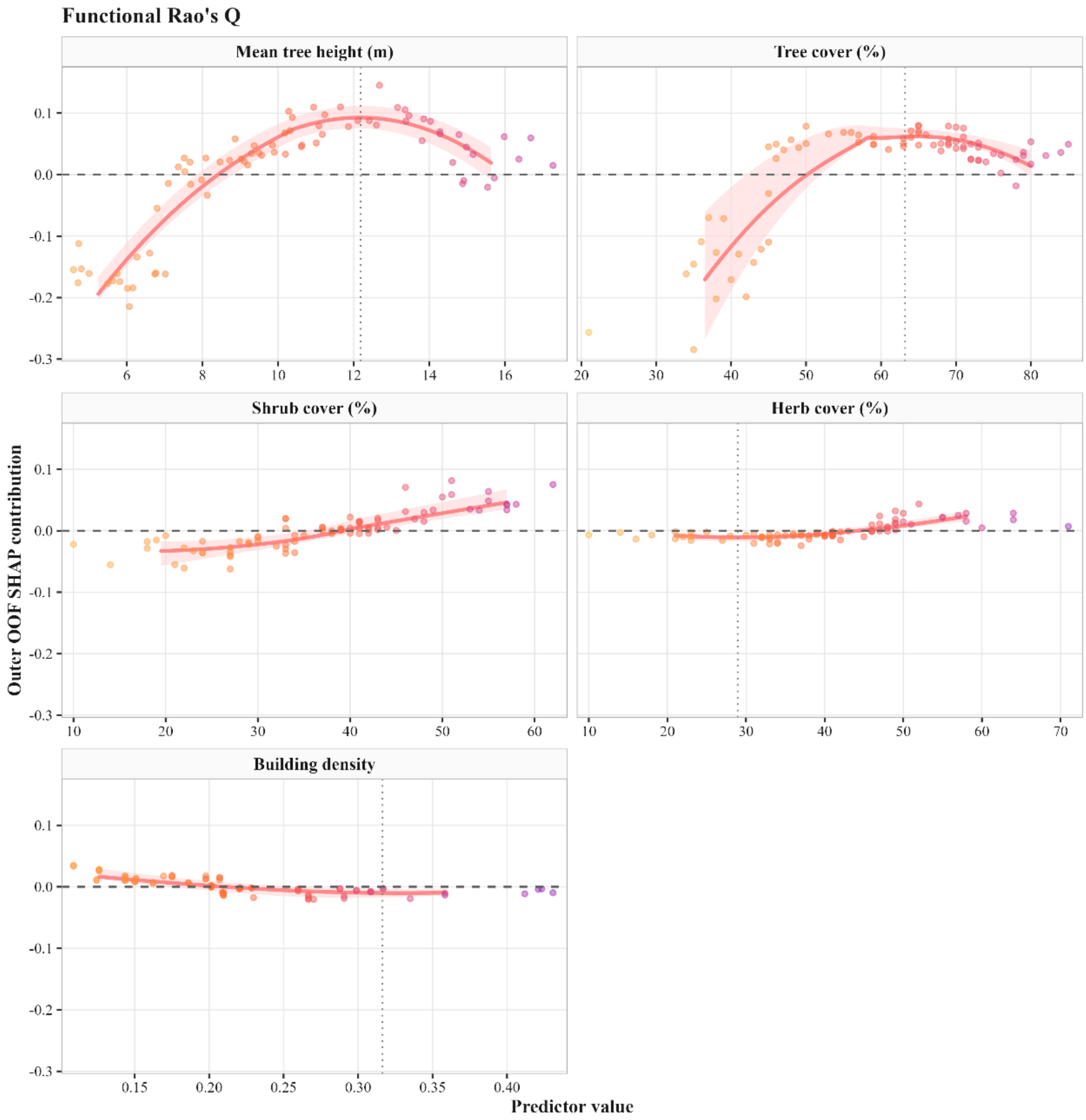

Figure S6. Selected OOF-SHAP responses for Visual FDis. Points show OOF-SHAP values; solid curves show LOESS-smoothed trends; shaded bands indicate smoothed 95% residential-area cluster-bootstrap intervals for binned median SHAP values; horizontal dashed lines indicate SHAP = 0; vertical dotted lines indicate candidate response turning locations.

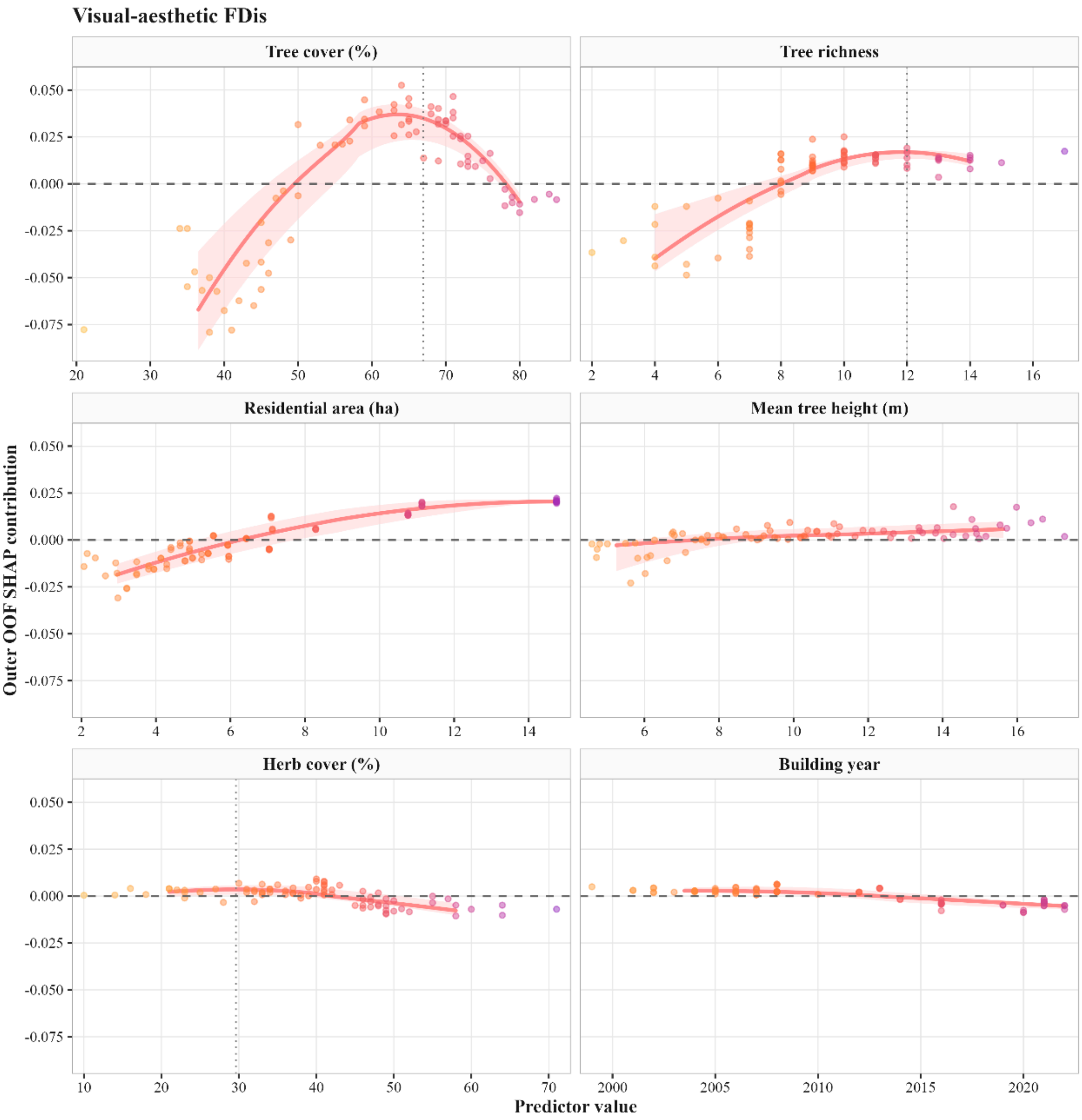

Figure S7. Selected OOF-SHAP responses for Visual Rao's Q. Points show OOF-SHAP values; solid curves show LOESS-smoothed trends; shaded bands indicate smoothed 95% residential-area cluster-bootstrap intervals for binned median SHAP values; horizontal dashed lines indicate SHAP = 0; vertical dotted lines indicate candidate response turning locations.

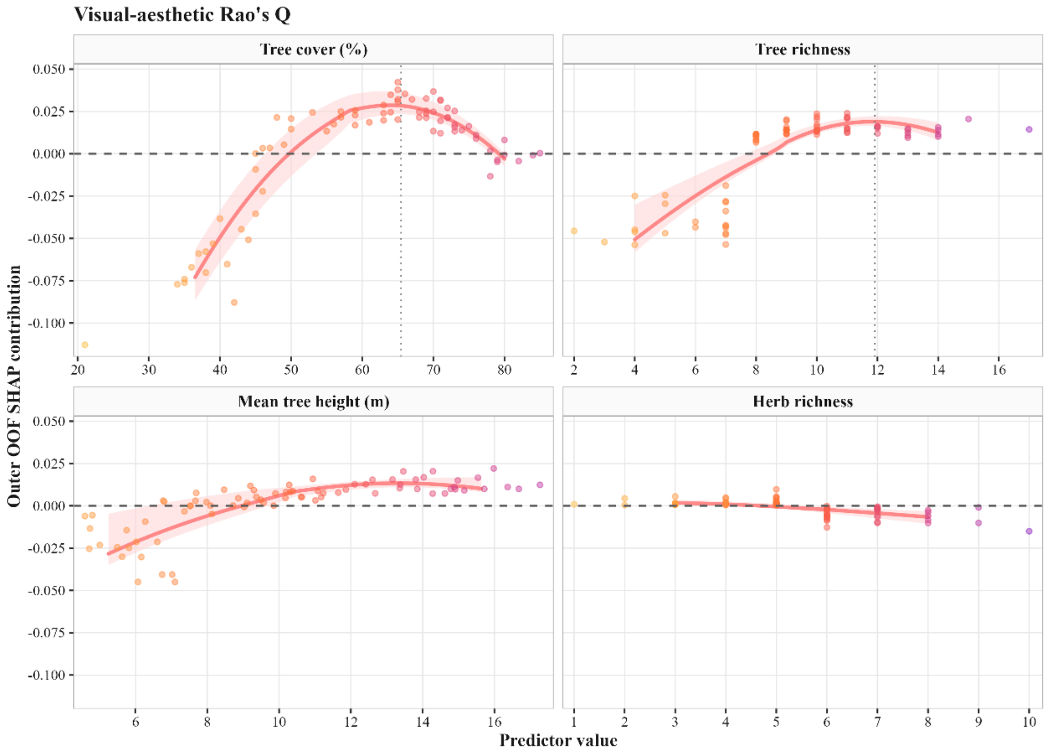

Figure S8. Functional–visual associations across all metric pairs. Points and horizontal lines show Spearman correlations and 95% cluster-bootstrap confidence intervals. Circles represent raw Spearman correlations, and diamonds represent partial Spearman correlations controlling for bird species richness and cumulative bird observation counts.

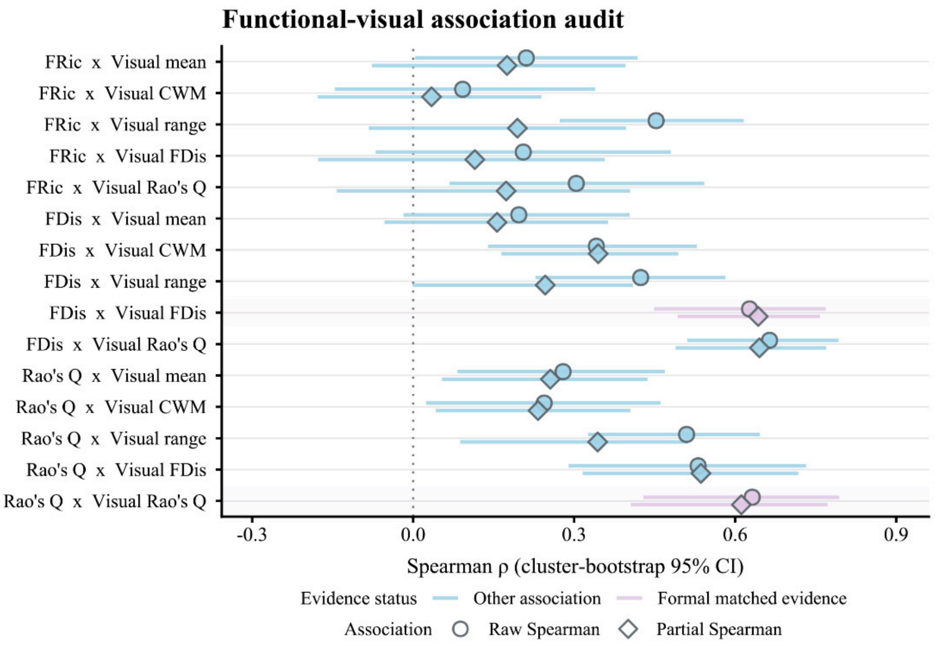

Figure S9. Functional–visual common environmental responses and local response divergence across environmental predictors. Symbol size represents joint relative contribution, and symbol shape indicates response class.

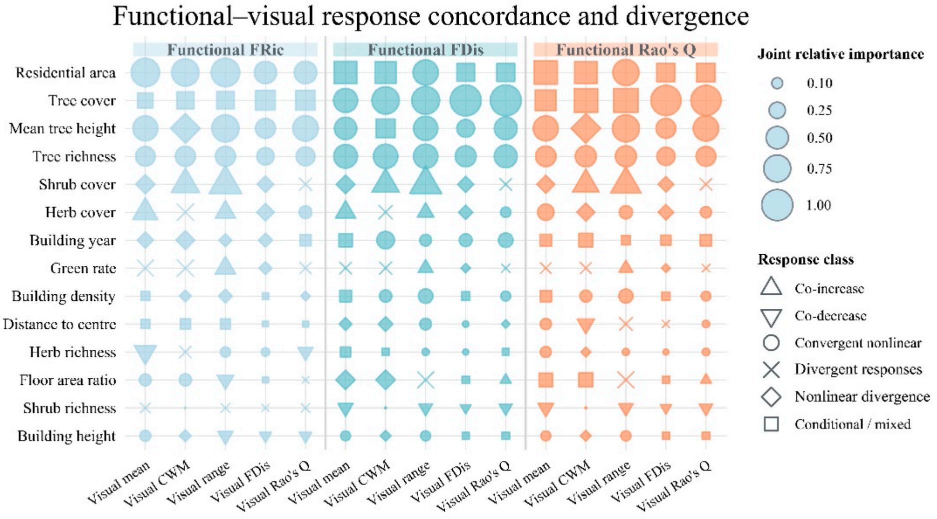

Figure S10. Shared-community-composition null distributions for the matched FDis and Rao's Q associations based on 4,999 frequency-constrained permutations of species-level visual scores. Dashed lines indicate observed correlations, and dotted lines indicate the 95% null intervals. Conditional XGBoost null intervals for response-curve correlations and joint relative contributions are summarized in Table S4.

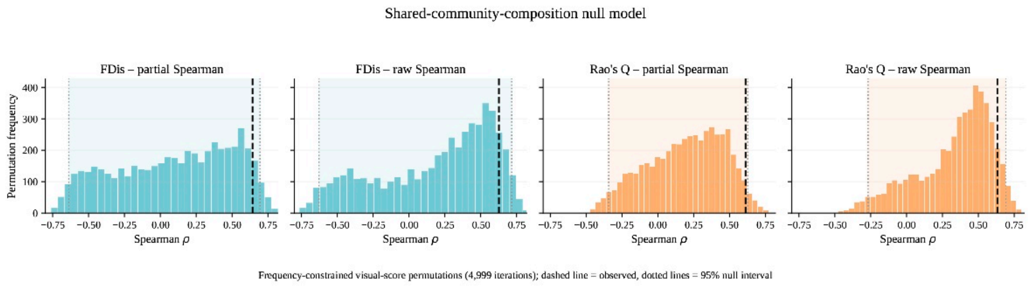

Figure S11. Robustness of functional–visual response classification to alternative rule settings (a) and leave-one-residential-area-out analysis (b). Point size indicates joint relative contribution.

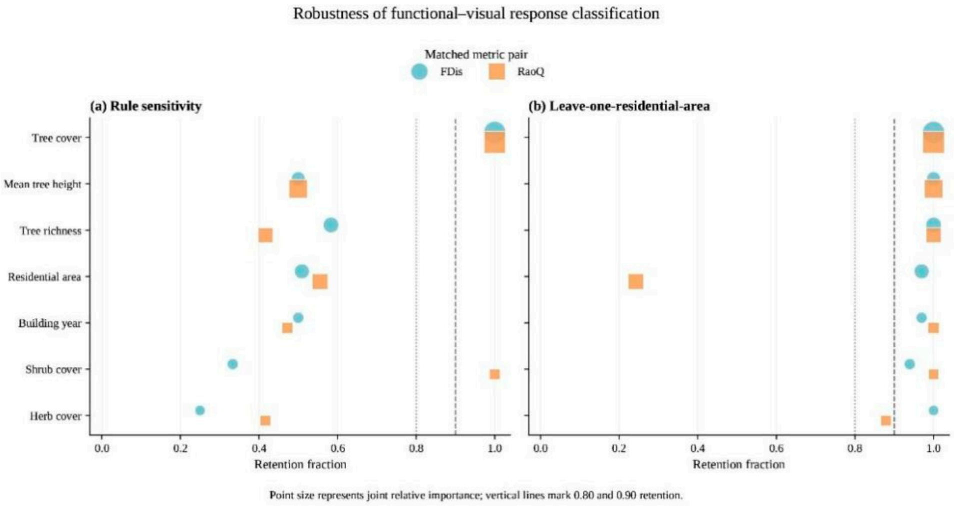

Figure S12. Sensitivity of OOF-SHAP response curves to alternative species-weighting schemes. Curves compare the original annual, square-root, presence/absence, and resident-only schemes for the four core diversity metrics along total tree cover, mean tree height, and tree richness gradients. SHAP values were normalized by the standard deviation of the corresponding response metric to facilitate comparison across weighting schemes. Previously calculated conditional response curves were evaluated over shared predictor ranges using the selected linear or three-degree-of-freedom natural-spline representation. The curves are descriptive model interpretations rather than causal effects or ecological thresholds. In particular, presence/absence-weighted visual models had negative OOF R^2 values, and their response curves should not be interpreted as reliable management relationships.

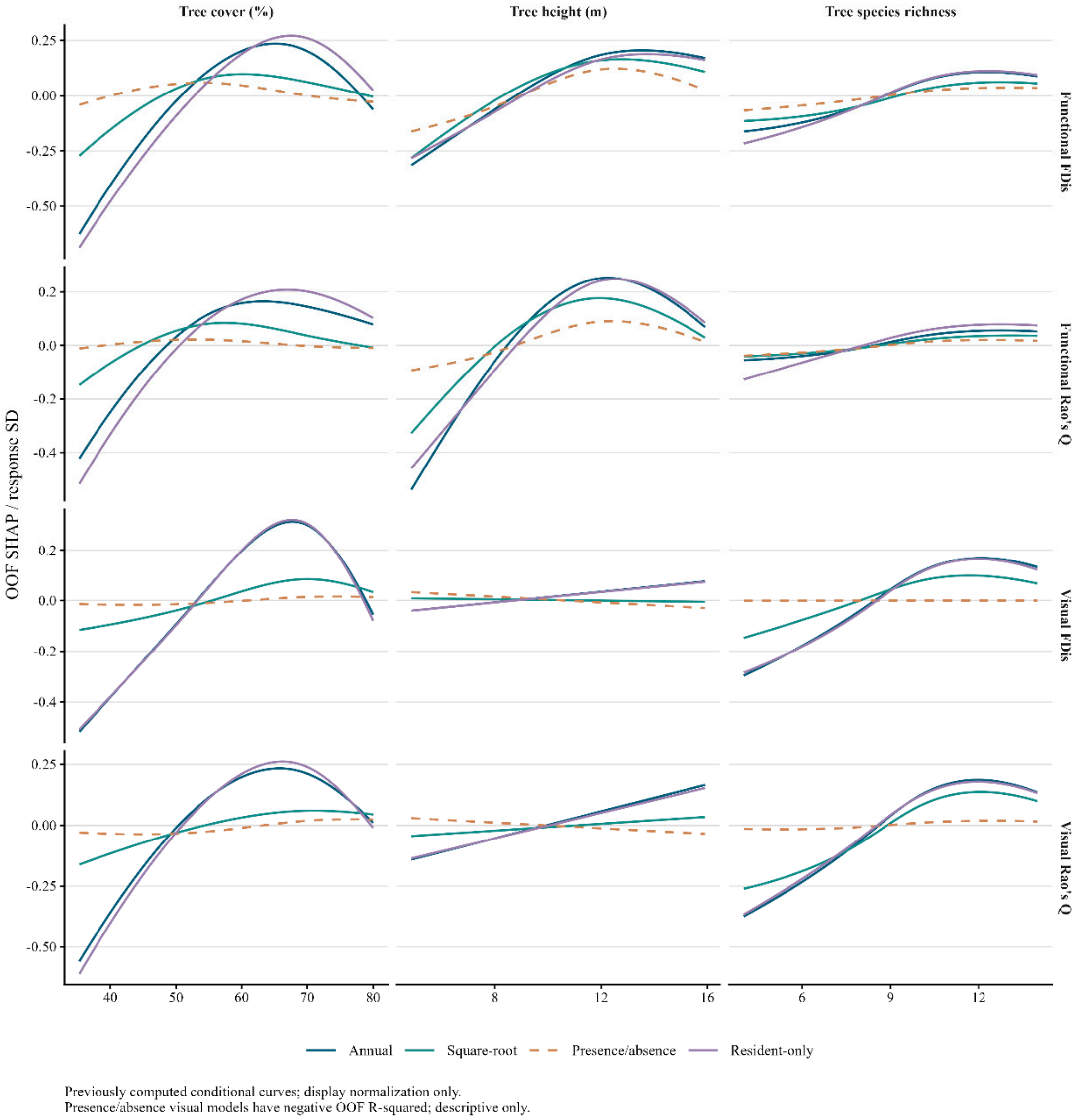

Supplement: Supplementary file 1 [file biology-15-01972-s001.zip › biology-4630561-supplementary.pdf]
